# Discrete modeling for integration and analysis of large-scale signaling networks

**DOI:** 10.1371/journal.pcbi.1010175

**Published:** 2022-06-13

**Authors:** Pierre Vignet, Jean Coquet, Sébastien Auber, Matéo Boudet, Anne Siegel, Nathalie Théret

**Affiliations:** 1 Univ Rennes, Inserm, EHESP, Irset, UMR S1085, Rennes, France; 2 Univ Rennes, Inria, CNRS, IRISA, UMR 6074, Rennes, France; 3 IGEPP, Agrocampus Ouest, INRAE, Université de Rennes 1, Le Rheu, France; Ecole Normale Supérieure, FRANCE

## Abstract

Most biological processes are orchestrated by large-scale molecular networks which are described in large-scale model repositories and whose dynamics are extremely complex. An observed phenotype is a state of this system that results from control mechanisms whose identification is key to its understanding. The Biological Pathway Exchange (BioPAX) format is widely used to standardize the biological information relative to regulatory processes. However, few modeling approaches developed so far enable for computing the events that control a phenotype in large-scale networks.

Here we developed an integrated approach to build large-scale dynamic networks from BioPAX knowledge databases in order to analyse trajectories and to identify sets of biological entities that control a phenotype. The Cadbiom approach relies on the guarded transitions formalism, a discrete modeling approach which models a system dynamics by taking into account competition and cooperation events in chains of reactions. The method can be applied to every BioPAX (large-scale) model thanks to a specific package which automatically generates Cadbiom models from BioPAX files.

The Cadbiom framework was applied to the BioPAX version of two resources (PID, KEGG) of the Pathway Commons database and to the Atlas of Cancer Signalling Network (ACSN). As a case-study, it was used to characterize sets of biological entities implicated in the epithelial-mesenchymal transition. Our results highlight the similarities between the PID and ACSN resources in terms of biological content, and underline the heterogeneity of usage of the BioPAX semantics limiting the fusion of models that require curation. Causality analyses demonstrate the smart complementarity of the databases in terms of combinatorics of controllers that explain a phenotype. From a biological perspective, our results show the specificity of controllers for epithelial and mesenchymal phenotypes that are consistent with the literature and identify a novel signature for intermediate states.

## Author summary

The computation of sets of biological entities implicated in phenotypes is hampered by the complex nature of controllers acting in competitive or cooperative combinations. These biological mechanisms are underlied by chains of reactions involving interactions between biomolecules (DNA, RNA, proteins, lipids, complexes, etc.), all of which form complex networks. Hence, the identification of controllers relies on computational methods for dynamical systems, which require the biological information about the interactions to be translated into a formal language. The BioPAX standard is a reference ontology associated with a description language to describe biological mechanisms, which satisfies the Linked Open Data initiative recommendations for data interoperability. Although it has been widely adopted by the community to describe biological pathways, no computational method is able of studying the dynamics of the networks described in the BioPAX large-scale resources. To solve this issue, our Cadbiom framework was designed to automatically transcribe the biological systems knowledge of large-scale BioPAX networks into discrete models. The framework then identifies the trajectories that explain a biological phenotype (e.g., all the biomolecules that are activated to induce the expression of a gene).

Here, we created Cadbiom models from three biological pathway databases (KEGG, PID and ACSN). The comparative analysis of these models highlighted the diversity of molecules in sets of biological entities that can explain a same phenotype. The application of our framework to the search of biomolecules regulating the epithelial-mesenchymal transition not only confirmed known pathways in the control of epithelial or mesenchymal cell markers but also highlighted new pathways for transient states.

## Introduction

The identification of genomic signatures associated with phenotypes and/or pathologies is an important goal in bioinformatics. Today, most genomic signatures are defined as sets of genes/markers whose transcriptional activation characterizes a phenotype. While these signatures are useful for diagnostic purposes, they do not provide information about genes potentially involved in the emergence or control of a phenotype, e.g., upstream controllers, and may therefore fail to identify potential therapeutic targets. Moving from diagnostic to causal signatures (i.e., sets of biomolecules that explain a phenotype) is not trivial due to the complex nature of controllers acting in competitive or cooperative combinations. More precisely, the causal signature contains all controllers and trajectory entities that are activated during the dynamical simulation of the model leading to the phenotype.

The first computational cornerstone is the search for causal signatures based on molecular interaction networks created without *a priori*, in order to avoid bias in the selection of knowledge to be included in the models. Indeed, most computational dynamical models are built using a manual selection of molecules and reactions, which limits the size of the models studied and may bias the analysis towards known molecules instead of exploring all the knowledge in the numerous existing databases [1].

The second computational cornerstone of identifying causal signatures is the design of methods for computing the controllers of the dynamical response of complex networks combining transcriptional regulation processes, signalling networks and metabolism. Although a wide variety of dynamical modeling formalisms have been developed using either deterministic, stochastic or logical approaches [2–5], the choice of a formalism must be adapted to the nature of the data and the biological question. When the goal is to identify controllers in as large a network as possible, formalisms based on discrete dynamical systems are the most appropriate, although they may have difficulties in capturing all the quantitative dynamical properties related to controls and competitions between molecular transformations.

In this paper, we argue that these two challenges can be addressed simultaneously through the Cadbiom framework. This approach relies on an expressive logic formalism using guarded transitions, an extension of Petri nets for modeling complex control events [6], to identify the controllers of large-scale molecular interaction networks. These networks are built automatically without any *a priori*, from standardized knowledge sources formalized in the BioPAX (Biological Pathway Exchange) format [7].

BioPAX is a reference ontology used for the systems biology domain, which is associated with a description format. It consists of classes specifically designed to represent biological pathways at the molecular and cellular level and to facilitate the sharing of biological entity metadata extracted from Uniprot, Rhea, pubmed or other sources. Our choice to focus on the BioPAX standard is motivated by its ability to describe, in an unified formalism, multiple layers of biological interactions (transformation, regulation and control processes) that are usually handled in separate and partially compatible formats such as the SBML [8], SBML-qual [9] or SBGN-ML formats [10]. To our knowledge, although each format has been successfully used to describe and analyse qualitative and quantitative properties of biological pathways, no approach is available to analyze and compare resources which are not encoded in similar formats.

For instance, the Pathway Commons database is a set of 23 databases supporting BioPAX format and describing 4,700 signalling and regulatory pathways, representing 2.3 million interactions [11], whose biological entities are linked to other biological resources. It integrates information from databases such as the Pathway Interaction Database (PID) [12] and the Kyoto Encyclopedia of Genes and Genomes (KEGG) [13].

Although all these databases are described according to the same ontology, combining them is actually difficult due to the diversity of their content in terms of biological compounds and reaction types. To overcome this limitation, a first strategy is to interpret the content of BioPAX resources as interaction graphs [14]. However, this prevents any dynamical analysis on the models. A second strategy is to extract specific, non-exhaustive information from the datasources, as done in the Omnipath framework [15], which uses conversion rules to extract only binary interactions from a subset of the BioPAX databases, which can then be manually enriched with generic Boolean rules [16]. All of these initiatives show that combining BioPAX data sources to analyze their combined dynamics is currently out of reach. Nevertheless, a key step towards this goal is to develop methods to compare the dynamics of BioPAX models. To this ends, the BioASF framework [17] relies on Petri nets to simulate BioPAX models. However, it requires defining the simulation rules manually and this approach can only be applied to small-scale models.

The goal of the present paper is to show that the Cadbiom framework [6], a dynamical abstraction based on guarded transitions [18], can be used to allow the identification and the comparison of causal regulators in large-scale models, formalized in the BioPAX language.

Initially, the Cadbiom tool [6] was designed to analyze the large-scale PID signalling network and to explore the causal effects of TGF*β* associated molecular compounds. We have very significantly refactored the Cadbiom approach to interpret all regulation, transformation and control processes of the BioPAX ontology into a single dynamical framework, which integrates the concepts of consumption, production, control and competition between resources. Applied to the PID, KEGG and ACSN databases, our approach has allowed us to extract and curate the relevant information from the BioPAX description of the databases in order to interpret them in a unified dynamical model and to compare their content despite their initial heterogeneity. The comparison of the resulting model structures highlighted their high level of complementarity. In addition, Cadbiom explores the dynamical model and identifies the controllers with respect to an expected phenotype (e.g., the activation of a gene). This allows for a comparison of the context of the databases at a dynamical level, through the comparison of controllers. As a biological application, we searched for putative regulators of genes that characterize the Epithelio-Mesenchymal Transition (EMT), a critical process in tumor progression whereby epithelial cells transdifferentiate into mesenchymal cells characterized by motility features [19, 20]. Together our data show specific patterns for the regulation of epithelial or mesenchymal marker genes and demonstrate how the combination of patterns provides new signatures for intermediate states of EMT.

## Materials and methods

### Data sources in BioPAX format

BioPAX (Biological Pathway Exchange) is a standard format that aims to enable the integration, exchange, visualization and analysis of biological pathway data to facilitate the understanding of complex biological processes.

The BioPAX ontology consists of classes specifically designed for use in systems biology, representing metabolic and signalling pathways, molecular and genetic interactions and gene regulatory networks. This ontology is compliant with the Linked Open Data initiative. The knowledge representation uses the Resource Description Framework (RDF) standard, relying on typed relations between entities, themselves typed using a controlled vocabulary associated with an ontology based on constraints and hierarchies between classes.

Pathway Commons is a web resource for biological pathway data [11] that integrates numerous databases of molecular interactions in several formats including the BioPAX format. We performed a systematic analysis of the content of two BioPAX models corresponding to the Pathway Interaction Database (PID) [12], and the Kyoto Encyclopedia of Genes and Genomes (KEGG) [13] database. We used the PID V9 and KEGG V10, each file being freely downloadable on the Pathway Commons website at https://www.pathwaycommons.org/archives/PC2.

We also used the Atlas of Cancer Signalling Network (ACSN) database [21] that is available as a catalog of 13 molecular maps formalized in the SBGN-ML format. Each map refers to biomolecule and reaction identifiers that are specific to each file. The maps are curated to homogenize the identifiers. The list of unified identifiers is then used to curate each individual map, so that the identifiers are prefixed by the name of the map to which they belong. The curated maps are converted separately to BioPAX format (OWL files) using the Cytoscape BiNoM plugin [22]. The files are imported into a local triplestore (Virtuoso server 07.20.3217) in a single graph. Finally, the BioPAX hierarchy of BiNoM generated objects is curated by removing the unnecessary RDF triples. Indeed, this tool defines the whole hierarchy of parent classes in each object instead of letting SPARQL queries to infer it via the RDFS reasoner. Details are provided on the biopax2cadbiom website http://cadbiom.genouest.org/doc/biopax2cadbiom/troubleshooting.html#conversion-issues.

### Methods to curate BioPAX models

The three BioPAX models were curated according to the following principles in order to homogenize and curate the databases (see details in S1 Appendix):

- We re-focused the analysis of the ACSN database to entities having the types Proteins, Complexes and SmallMolecules and their associated interactions.
- We removed classes (generic entities) and nested classes when they were not involved in BioPAX reactions or controllers. When the deletion took place, the member entities inherited the properties ModificationFeatures and Location from their parent class.
- We processed the databases to group all duplicated entities together, so that each group of similar entities eventually appeared in the processed model as a single entity with a unique identifier.
- The controls of type Modulation that are present at low frequency were not considered.
- We noticed that all databases contain biomolecules playing both the roles of product and reagent of the same Interaction type. We considered them as catalysts, and therefore removed them from the list of reactants to create new objects Catalysis regulating the concerned reactions.

### From BioPAX models to guarded transition models

The main principle of the approach introduced in this paper is to interpret the BioPAX models into Cadbiom models, which are discrete dynamical models consisting of biological entities linked by guarded transitions [6]. A guarded transition *t* is defined by a quadruplet (*A*, *B*, *h*, Cond) and denoted by *t*: *A*^*h*[*Cond*]^ → *B*. The *input* *A* and the *output* *B* of the transition are biological entities or biomolecules. The condition *Cond* is a logical formula with the logical operators “and”, “or” and “not” and the biomolecules as variables. *h* is an *event* allowing to model the series of time-clock when the guarded transition is activated.

**Fig 1:**
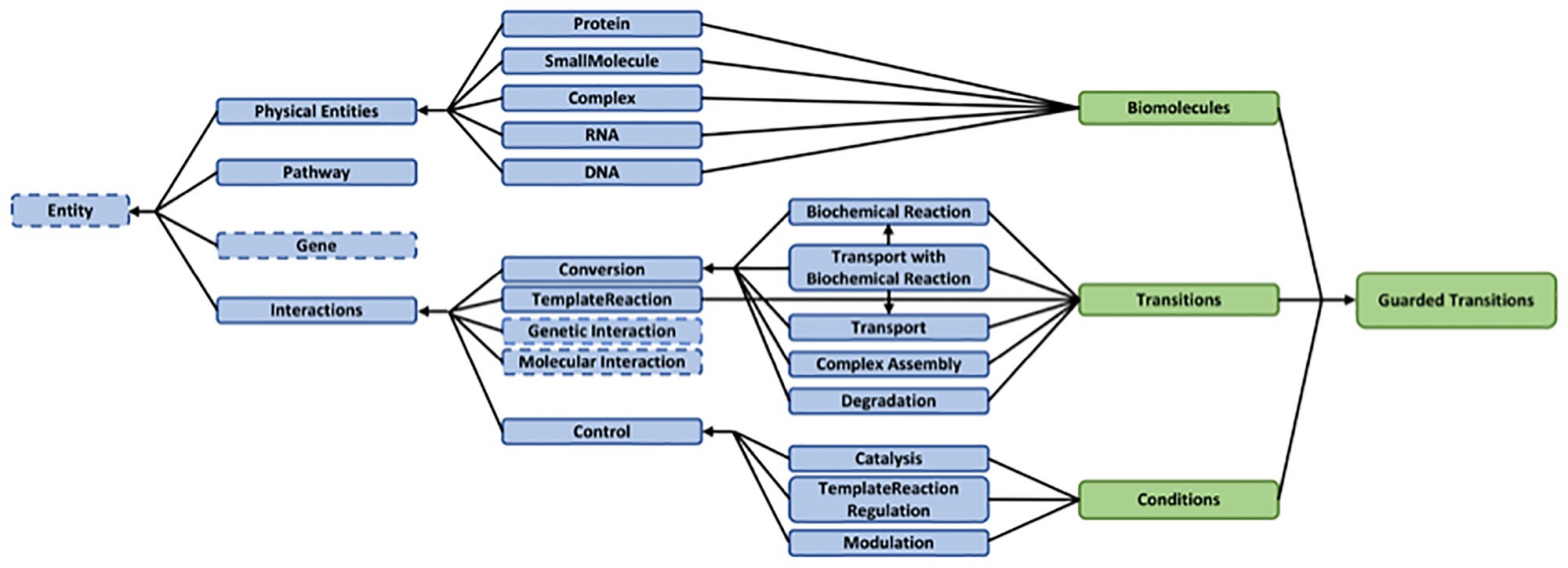
Conversion of BioPAX classes into guarded transitions. BioPAX classes (in blue) denoting cellular and molecular objects in biological pathways are interpreted as Cadbiom entities, transitions and conditions (in green). Unused BioPAX classes are surrounded by dashes.

As shown in Fig 1, the guarded transition formalism is compliant with the BioPAX format. Indeed, most BioPAX classes can be naturally interpreted as *entities*, *transitions* or *conditions* in the formalism of guarded transitions. Among the derivatives of the Entity mother class that is the root of the ontology, Cadbiom manages the PhysicalEntity and Interaction classes. All instances of the PhysicalEntity mother class and its subclasses (Protein, SmallMolecule, Rna, Complex, Dna) are interpreted as Cadbiom *biomolecules*.

The BioPAX Interaction class gathers all biochemical processing involving biomolecules. All instances of the TemplateReaction class and the Conversion class, which includes the BiochemicalReaction, ComplexAssembly, Transport, Degradation and TransportWithBiochemicalReaction subclasses, are interpreted as Cadbiom *transitions* (see details in the Results section). The MolecularInteraction and GeneticInteraction subclasses are not considered by the Cadbiom formalism because they cannot be interpreted as controlled biochemical reactions.

Finally, the Control subclass of the Interaction class is used to describe the regulation of biological processes by entities or by other processes. This class is parent of Catalysis, TemplateReactionRegulation and Modulation. The Catalysis class is used to describe enzymatic reactions; it refers to a PhysicalEntity controller and a controlled Interaction, which is either a Conversion or a TemplateReaction. The Modulation class describes the regulation of enzymatic reactions. All their instances are interpreted as *conditions* in the Cadbiom formalism.

**Fig 2:**
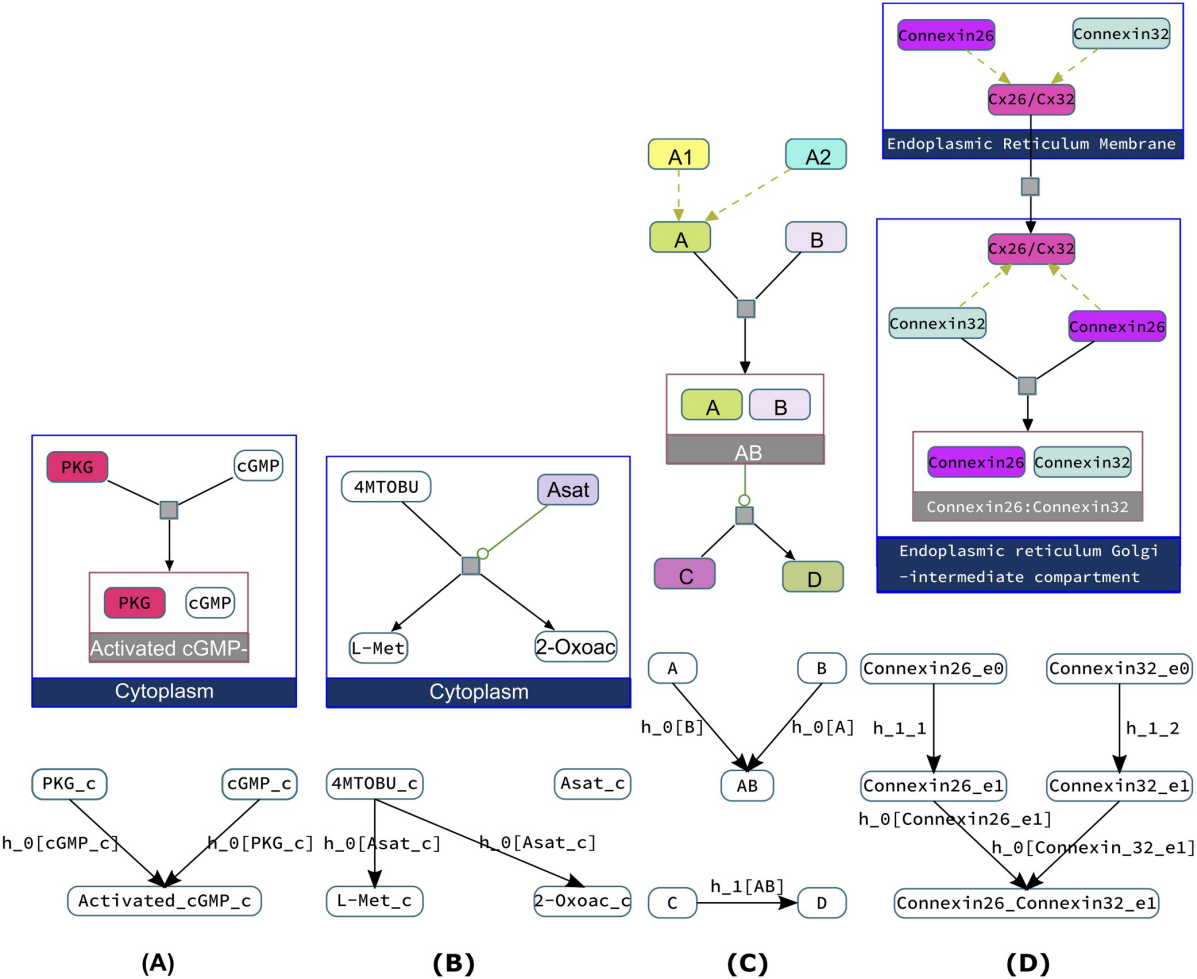
Interpretation of BioPAX models (represented in colored CheBi format) in guarded transitions. (A) The complex assembly of PKG-cGMP is rewriten by two guarded transitions linked by the common event *h*_0_. Each guarded transition has one of the substrates as *input* and the other substrate as *condition*. By introducing a common *h*_0_ event in their guards, we model the fact that both guarded transitions must be activated simultaneously to produce the complex. This can happen if and only if, both *inputs* are present and if the condition guards are satisfied, i.e., if PKG and cGMP are present. (B) The catalysis reaction that decomposes the compound 4MTOBUT into L-Met and 2-Oxoacid under the regulation of Asat is modeled by two guarded transitions, sharing the same event *h*_0_ and the same Asat condition guard. (C) In the generic case when a BioPAX entity class *A* consists of two entities *A*1, *A*2 which do not appear individually in any reaction in the model, the entity class *A* is conserved as a single compound *A* in the guarded transition model, and *A*1 and *A*2 are eliminated from the Cadbiom model. The complex assembly reaction between any element of the class *A* and another compound *B* is modeled by two transitions producing the entity *AB*. This entity is then used in the guard of the transition modeling the biochemical reaction transforming *AB* into *C* and *D*. (D) A transport reaction of the Connexin26 and Connexin32 molecules, gathered in the class *Cx*26/*Cx*32, from *Endoplasmic reticulum membrane* to *Endoplasmic reticulum-Golgi intermediate compartment*. In the latter compartment, a complex assembly occurs between the connexins. In the guarded transition model, the class *Cx*26/*Cx*32 is deleted. The compounds Connexin26 and Connexin32 are each duplicated into the two compartments. The transport of each compound is modeled by independent guarded transitions.

We implemented a complex rewriting strategy inspired by the seminal paper [6] to automatically interpret any BioPAX model into a guarded transition model. This strategy is based on three principles: interpret BioPAX physical entities to biomolecules and genes in Cadbiom models, then interpret interactions to Cadbiom guarded transitions and finally expand classes according to the Interaction context. This strategy is illustrated on few examples in Fig 2. Details are given in S1 Appendix.

### Dynamics of guarded transition models

We use the formalism of guarded transition to analyze the dynamics of Cadbiom models according to a non-deterministic framework. The formalism is inspired by the UML state transition semantics enriched by an algebra (see S1 Appendix for details).

According to this formalism, we highlight four concepts of nodes of interest with the analysis of the dynamics of a Cadbiom model. The first one is related to the complete Cadbiom model whereas the three other ones are related to phenotypes. All detailed definitions are given in S1 Appendix.

- *Boundary entities of the* Cadbiom *model* (named “frontier places” in the seminal paper [6]) are all entities of the full cadbiom model which are not the output of guarded transitions.
- *The controllers of a phenotype* are all boundary entities of the Cadbiom model which are activated in at least one dynamical simulation of the model leading to the activation of the phenotype according to the guarded transition semantics.
- *The trajectory entities of a phenotype* are all intermediate entities of the Cadbiom model activated in at least one trajectory leading to the phenotype.
- *The* Cadbiom *signature of a phenotype* encompasses all controllers and trajectory entities of the phenotype according to the Cadbiom semantics. It is computed with a SAT-based approach, which recursively simulates the trajectories of the guarded transition model and computes the minimal set of entities forcing the entities to the expected phenotype as well as the intermediary states of the system before reaching the phenotype. Therefore, the causal signature contains all controllers and trajectory entities that are activated during the dynamical simulation of the model leading to the phenotype.

**Fig 3:**
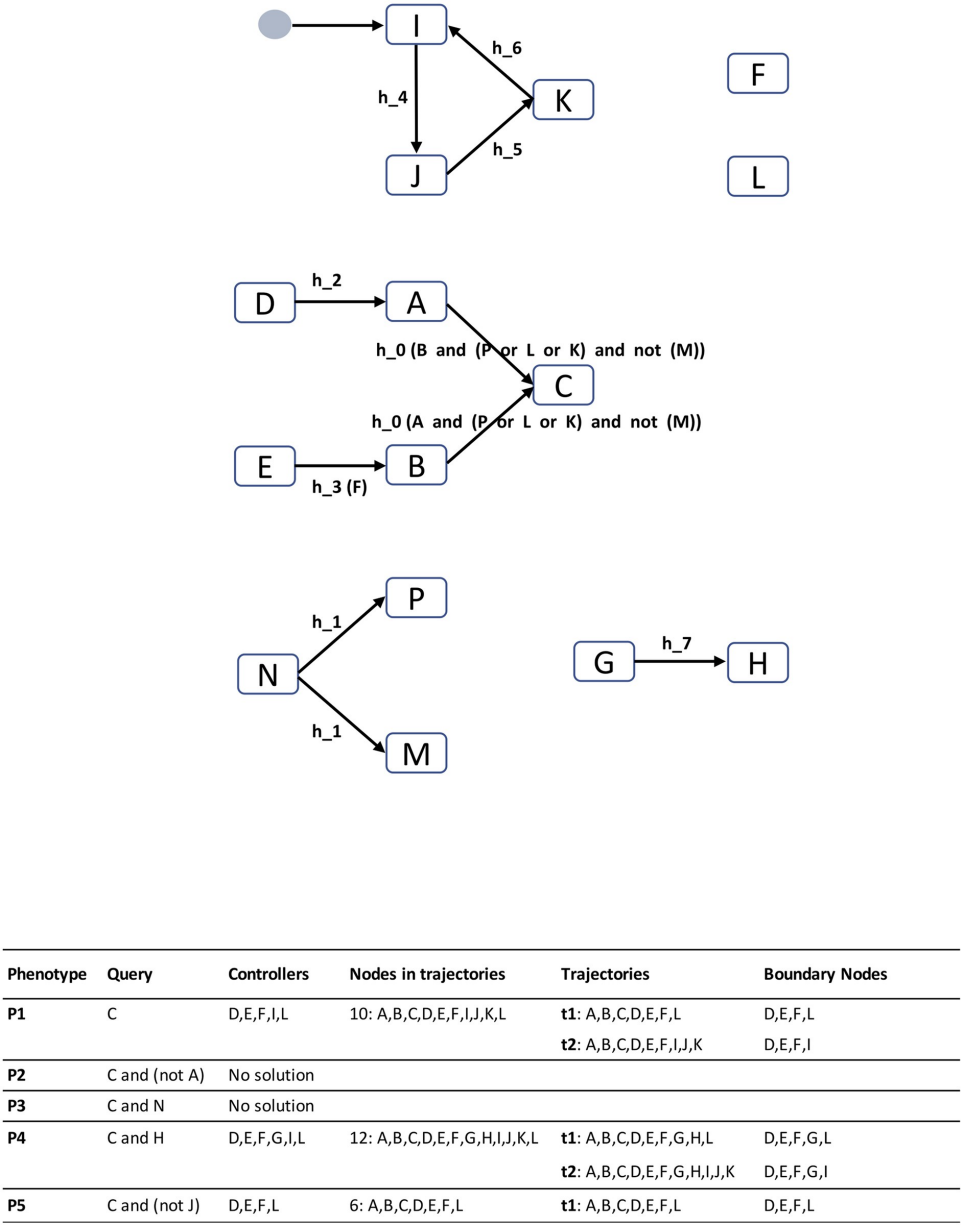
Example of a guarded-transition model and controllers associated with different queries. The guarded transition model consists of 15 biomolecules (*A*,*B*,*C*,*D*,*E*,*F*,*G*,*H*,*I*,*J*,*K*,*L*,*M*,*N*,*P*), 11 transitions (black arrows) for 7 temporal events (*h*_1_, … *h*_7_), which model reactions. Two events carry a guard (i.e., logical formula) restricting their triggering: *h*_0_ requires the satisfiability of the formula *A and (P or L or K) and not(M)*, while *h*_3_ requires the presence of the reagent *F*.*F* is considered to be an activator of *h*_3_, just as *A, P, L and K* are activators of *h*_0_ (i.e., in the presence of *A*, and one of the latter 3 is sufficient to trigger *h*_0_); in contrast, *M* is an inhibitor of *h*_0_. The event *h*_1_ consumes *N* to produce simultaneously *P* and *M*; it is never triggered in the context of obtaining *C* because of the production of the inhibitor *M*. A cycle of 3 biomolecules *I, J, K*, constitutes a strongly connected component. This cycle is resolved by arbitrarily adding a *virtual* node named *cycle_initiation_node* on the first of the lexicographically sorted nodes (i.e., *I*); this has the effect of adding *I* to the system boundary entities.

We illustrate the causality analyses in Fig 3 by detailing five queries performed on a toy model. The first query is related to the reachability of node C in the model (phenotype **P1**). The production of the compound requires both A, which in turn requires D, and B, whose production requires both the input E and the activator F. The transitions from A to C and from B to C are conditioned by the same event *h*_0_, the absence of M, and the presence of either P, or L or K. This latter condition is satisfied either when L is activated, or when the cycle I, J, K is activated. Therefore, there are two different trajectories allowing for the production of the compound C: the trajectory (A,B,C,D,E,F,L), where boundary entities are D,E,F,L and the trajectory (A,B,C,D,E,F,I,J,K), where boundary entities are D,E,F,I. Therefore, there are five (D,E,F,I,L) controllers of the phenotype corresponding to the activation of C.

When the expected phenotype **P2** is the activation of C with the absence of A (Query 2), the model analysis reports that there is no solution because A is required for the production of C. When the expected phenotype **P3** is the activation of C and N (Query 3), the model analysis also reports that no trajectory is possible, because the activation of C is conditional on the absence of M while the presence of N implies the production of M. Verification of the query “C and H” (Phenotype **P4**) is possible by adding the transition from G to H to the solutions in Query 1. Verification of the query “C and not J” (Phenotype **P5**) no longer allows the use of the trajectory relying on the I,J,K cycle.

Subsequently, the search for sets of controllers and trajectory entities to describe and reach any state of the system can be considered as a verification problem on Boolean variables. These problems are solved using the *cadbiom-core* package implemented in the Cadbiom framework (see below).

### The Cadbiom framework

The Cadbiom framework and its suite (GNU GPL License) is written in Python and hosted on the PyPI (Python Package Index) platform for easy deployment. The installation procedure, documentation for users and developers, sources and examples illustrating the interpretation processes of large-scale databases are detailed at http://cadbiom.genouest.org/doc/cadbiom/index.html and in S1 Appendix.

In order to adapt to large-scale model analysis, the former library developed in [6] was modified as follows: (i) time-consuming functions are rewritten in C language, (ii) an architecture implementing multiprocessing technology is integrated into the command line module, (iii) the cryptominisat solver is upgraded. Overall, these changes increase the capabilities of Cadbiom such that the performance of the current implementation is 2000 times faster for the same query on a single CPU than the original implementation.

An important parameter of the Cadbiom framework is the maximum number of trajectories computed by the solver for each query. Prior to this study, we tested three values for this parameter (400, 900 and 5000) on a selection of genes. We noticed that extending the analysis to 900 trajectories did not significantly change the lists of trajectory entities compared to the computation of 400 trajectories. We therefore retained the value of 400 for the full study because it corresponded to the best compromise between the completeness of the trajectory computation and execution time analysis.

In addition, the biopax2cadbiom package was developed as a stand-alone module. It is compatible with the BioPAX Level 3 specifications, which offers the possibility to define entities under several states (including a generic state). The tool executes queries in the SPARQL query language to automatically create Python classes based on BioPAX specifications. Objects instantiated from these classes are then used in the translation operations of the BioPAX formalism to the Cadbiom format. Documentation is available at http://cadbiom.genouest.org/doc/biopax2cadbiom/index.html.

## Results

### Curation of PID, KEGG and ACSN BioPax models

We performed a systematic content analysis of 2 databases available on Pathway Commons, PID (cell signaling) and KEGG (metabolism and signaling reactions), as well as the ACSN database (Cancer signaling network), which are the largest reaction databases available in the BioPAX format. In order to homogenize the three databases, we performed some curation to handle the generic entities, the nested classes and the duplicated entities (see detail in S1 Appendix). The characteristics of the models are presented in Table 1. The effect of the curation processes is very limited; we observed a slight increase in the PID (11,124 vs 10,526) model and no changes in the KEGG and ACSN models.

**Table 1:** Makeup of the BioPAX models from three resources (PID, KEGG, ACSN). The BioPAX files of the three resources were parsed in order to identify Physical Entities, Controls (Catalysis and TemplateReactionRegulation) and Reactions (Conversion and TemplateReaction). For the ACSN resources, Physical Entities were curated in order to reanotate their types when possible. The curation processes focused on compressing duplicate entities and creating controls (with the type catalysis) for reactions where a reactant is also a product.

	PID	KEGG	ACSN
**PhysicalEntities in the BioPAX resource**	10,526	3,536	11,922
Entities with the generic type PhysicalEntity	0	0	0
Entities with the type Protein	6,194	1,872	6,851
Entities with the type Complex	4,137	0	2,323
Entities with the type SmallMolecule	173	1,664	554
Entities with the type Dna	0	0	1,030
Entities with the type Rna	22	0	1,164
Duplicated entities	699	135	74
Groups of duplicated entities	339	61	23
Generic entity classes	403	0	0
Used classes	228	0	0
Nested classes	23	0	0
Classes with ModificationFeatures	157	0	0
**PhysicalEntities after curation (handling duplicated entities and generic classes)**	11,124	3,536	11,922
**Controls in the BioPAX resource**	6,145	1,782	6,186
Controls with the generic type Control	322	0	0
Controls with the type Catalysis	3,800	1,782	6,186
Controls with the type TemplateReactionRegulation	2,023	0	0
Controls with the type Modulation	0	0	0
Interactions with similar entities as reagents and products	50	934	333
Classes in controls	102	0	0
**Controls after curation (introducing catalysing effects, removing modulation)**	6,195	2,716	6,519
**Conversions and TemplateReactions in the BioPAX resource**	6,504	1,786	9,305
Reactions with the type BiochemicalReaction	1,824	1,782	6,863
Reactions with the type ComplexAssembly	2,722	0	1,743
Reactions with the type TemplateReaction	1,492	0	0
Reactions with the type Transport	312	0	699
Reactions with the type MolecularInteraction	0	4	0
Reactions with the type Degradation	0	0	0
Reactions with the type TransportWithBiochemicalReaction	154	0	0
Proteins involved as reactants	3,360	0	4,840
SmallMolecules involved as reactants	128	1,585	0
Complexes involved as reactants	3,768	0	2,217
**Reactions after curation**	6,504	1,786	9,305

As shown in Table 1, the PID and ACSN BioPAX databases contain few SmallMolecules and the reactants of reactions are mainly Proteins and Complexes. This type of content is representative of signaling pathways with proteins involved in either signalling reactions or control of these reactions.

In contrast, the KEGG BioPAX file contains proportionally more SmallMolecules (1,585) than the other databases but does not contain ComplexAssembly reactions. There are as many BiochemicalReaction (1,786) as Control reactions (1,782) (only from the class Catalysis) suggesting that each reaction is effectively catalyzed by a single biomolecule. This is because the KEGG BioPAX file consists specifically of small molecules and corresponds to the metabolism part of the entire KEGG database, with no description of the signalling part.

### Interpreting BioPAX models into guarded transitions

The BioPAX format is compliant with the guarded transition formalism because BioPAX classes can be interpreted as entities, transitions or conditions. We used this mapping to implement a complex rewriting strategy inspired by the seminal paper [6] to automatically interpret any BioPAX model into a guarded transition model. The main principle introduced in [6] is to rewrite a reaction *r*: *A* + *B* → *C* + *D* controlled by the catalyzer *E* by four guarded transitions *t*_1_: *A*^*h*[*B*]^ → *C*, *t*_2_: *B*^*h*[*A*]^ → *C*, *t*_3_: *A*^*h*[*B*]^ → *D*, and *t*_4_: *B*^*h*[*A*]^ → *D*. Since all these guarded transitions share the same event *h*, they are linked by the constraint that they must be activated simultaneously. As detailed in the Material and Methods section, this principle was adapted to the content of each BioPAX database in order to capture their main specificities and homogenize their analyses.

The rewritting strategy was applied to the PID, KEGG and ACSN BioPAX models to construct three Cadbiom guarded transition models of large-scale regulatory and signalling networks. These Cadbiom models were obtained by using the package biopax2cadbiom to interpret BioPAX databases. The results of the conversions of the KEGG and PID databases extracted from Pathway Commons, and the ACSN database are available on the website http://cadbiom.genouest.org/doc/cadbiom/workflow_overview.html#prebuilt-models. As shown in Table 2, the Cadbiom models contained slightly fewer entities than the BioPAX models: there are 7%, 26% and 13% fewer entities in the PID, KEGG and ACSN Cadbiom models, respectively.

**Table 2:** Characteristics of the Cadbiom models obtained after the conversion of BioPAX sources into models with guarded transitions. The Cadbiom models are described by entities, events and transitions. They are compared to the numbers of entities and reactions in the BioPAX models. The boundary entities correspond to the peripherical entities of the model. They are described according to their type and their role in the cadbiom model.

Cadbiom **models**	PID	KEGG	ACSN
**Entities**	9,788	2,604	10,313
Ratios between Cadbiom and BioPAX entities	0.93	0.74	0.86
Gene entities	788	2	1,035
HUGO identifiers for gene entities	743	0	749
**Events**	7,501	1,570	8,819
Ratios between Cadbiom events and BioPAX reactions	1.22	0.88	0.95
**Transitions**	11,036	5,220	11,394
**Boundary entities of the model**	3,925	1,420	3,693
Genes	788	2	929
Rna	3	0	122
Proteins	2,021	1,016	2,179
Complexes	1,011	0	252
Small Molecules	83	402	132
Cycle initiation entities	19	0	79
Physical Entities (undefined)	0	0	0
**Role of boundary entities**			
Inputs of transitions	3,324	386	3,078
Members of conditions	2,442	1,387	1,902
Both Input of transitions and member of conditions	1,743	355	1287
Control (only members of conditions)	699	1,032	615

The ratios between the number of transitions in the Cadbiom model (see Table 2) and the number of reactions extracted from the BioPAX resource (see Table 1) are 1.69; 2.92 and 1.22 for the PID, KEGG and ACSN models, respectively. This suggests that formalization of biologic data involving entities is variable. The number of transitions depends on entities participating in one Cadbiom event and the ratio illustrates the mean number of entities implicated in a reaction. A Cadbiom event is close to the biochemical reactions in BioPAX syntax. Moreover we observed that the Cadbiom models of PID, KEGG and ACSN have a number of events similar to the number of reactions in the BioPAX models (+15.33%, -12.09%, -5.22%, respectively).

### Comparison of the Cadbiom models

**Fig 4:**
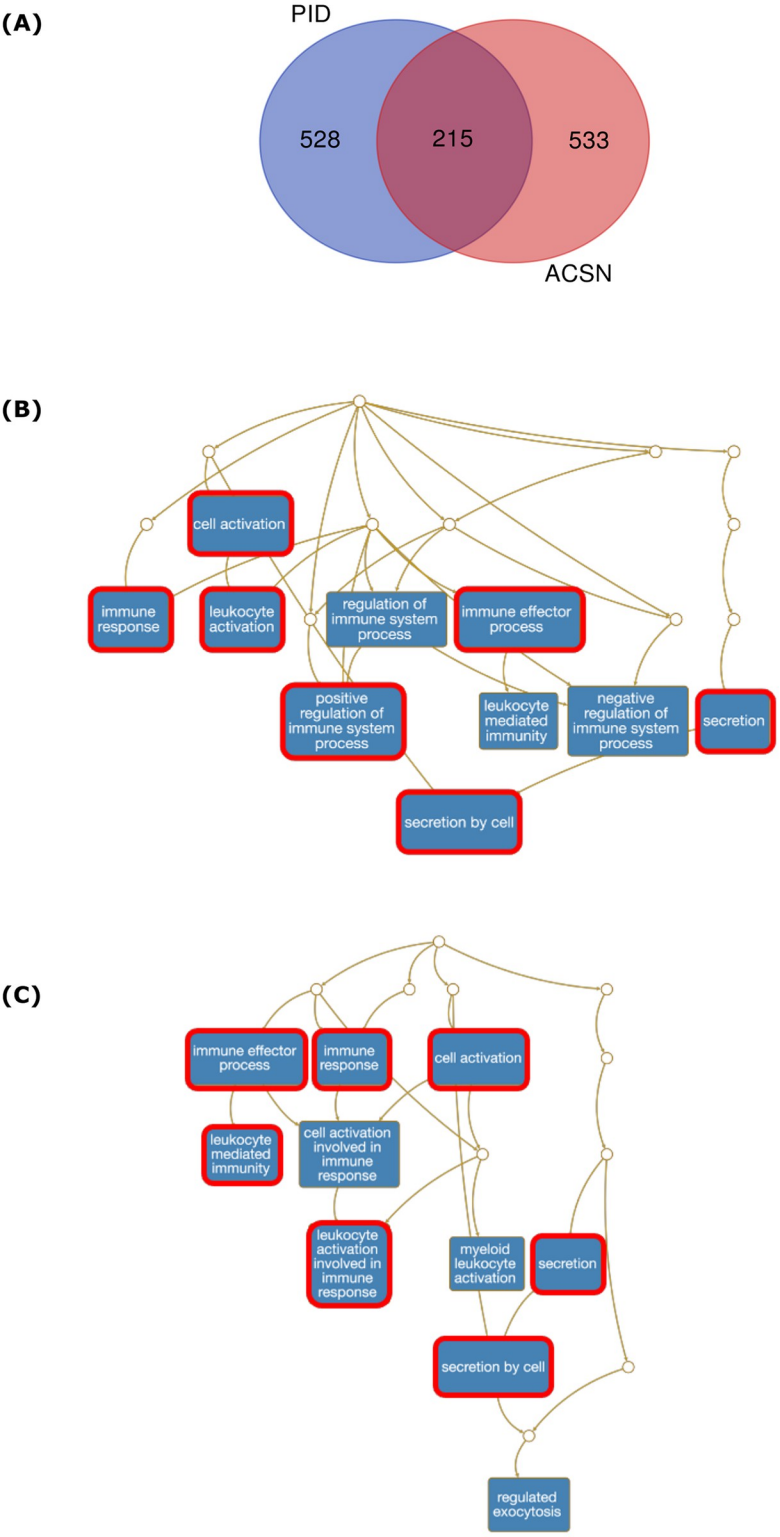
Comparative analysis of Hugo identifiers associated with gene entities of the PID and ACSN cadbiom models. (A) Venn diagram describing the intersection between the HUGO identifiers appearing in gene entities of the PID and ACSN cadbiom models. (B) Over-representation analysis of HUGO identifiers from PID. (C) Over-representation analysis of HUGO identifiers from ACSN. Red boxes are common GO terms between PID and ACSN models.

As shown in Table 2, we observed that the KEGG Cadbiom model contains only 2 genes which is consistent with the fact that KEGG contains only metabolism-related pathways. The PID and ACSN models have 788 and 1,035 gene entities, respectively, which are entities whose identifiers contains the string “_gene”. We noticed that some entity names may refer either to a same gene with various annotations (related to the location, states etc.) or to a gene family. We performed a manual curation of each gene entity to associate a HUGO identifier to each of them. Based on this curation, we identified 743 and 748 unique HUGO gene identifiers in the PID and ACSN models, respectively (S1 Table). Only 215 genes are shared by the two models suggesting specificities of each model (Fig 4A). To further compare these two models, we performed an over-representation analysis of Gene Ontology terms (Biological process) associated with these genes, using the WEB-based GEne SeT AnaLysis Toolkit [23]. As shown in Fig 4B, seven out of ten enriched GO terms are similar in these models demonstrating functional similarities between genes in the PID and ACSN models.

The comparison between ACSN and PID database contents has been previously published [21] and the authors concluded to a low overlap though the most canonical molecular pathways are represented in both PID and ACSN databases. Consistent with this, while the comparison between PID and ACSN Cadbiom models showed similar enriched GO terms, we observed differences in specific pathways such as the leptin signaling pathway, the IL27RA signaling pathway, the IL23R signaling pathway, the Neurotrophic factor mediated Trk signaling pathway and the Circadian rhythm pathway which are present in the PID model but absent in the ACSN model (see details in S1 Appendix). On the other hand, some specific processes were enriched in ACSN compared to PID, such as the senescence network which is not specifically documented in PID. It is difficult to compare an “ACSN module” with a PID pathway because the module gathers more information. For example, the canonical WNT module in ACSN contains 200 proteins while the WNT signaling pathway in PID contains only 28 proteins.

To better compare the PID and ACSN Cadbiom models, we extracted information about peripherical entities of the models. To this end, we defined as *boundary entity* of a Cadbiom model every node that is not the output of any transition in the model. In other words, there is no way to produce these entities with reactions in the model. In the following section, it will be assumed that they are activated in the initial states of the dynamical model simulations. The classification of boundary entities by type and by role played in transitions is detailed in Table 2 and illustrated in Fig 1 in S1 Appendix. The PID and ACSN models have similar number of boundary entities (3,925, 3,693) and the number of boundary entities relative to the total number of entities is consistent between the models (PID: 0.40, ACSN: 0.36). Because boundary entities are not produced by any reaction of the model, the ratio of boundary entities to total entities illustrates the structure of the models. Consistent with this, we hypothesize that metabolic reaction chains are structurally different from signaling reaction cascades and could explain that KEGG is the smallest model since its boundary entities/total entities ratio is 0.54.

Among the boundary entities, the distribution of genes, proteins and small molecules are similar between the PID and ACSN models (genes; 20% and 25%, respectively; proteins: 51% and 59%, respectively and small molecules: 2% and 3.5%, respectively). The PID model contains very few RNA boundary entities, which is explained by the fact that the PID model contains only 3 Rna entities in total. The ACSN model contains 122 Rna boundary entities, which is 11% of the total number of Rna contained in the model. It is important to note that the number of complexes among PID boundary entities is four times higher than in the ACSN model (26% and 7%, respectively). This suggests that biological reactions describing complex formation are more detailed in the ACSN model than in the PID model, implying that the complex entities in the ACSN model are considered as intermediate biomolecules in pathways described in the model while the complex entities in the PID model are considered as initiating biomolecules of its pathways.

Analysis of the KEGG model data confirms that it describes only metabolic processes: in this model built from the Pathway Commons repository, all proteins are boundary entities of the model, suggesting that they play the role of controllers of enzymatic reactions rather than components of signaling networks.

**Fig 5:**
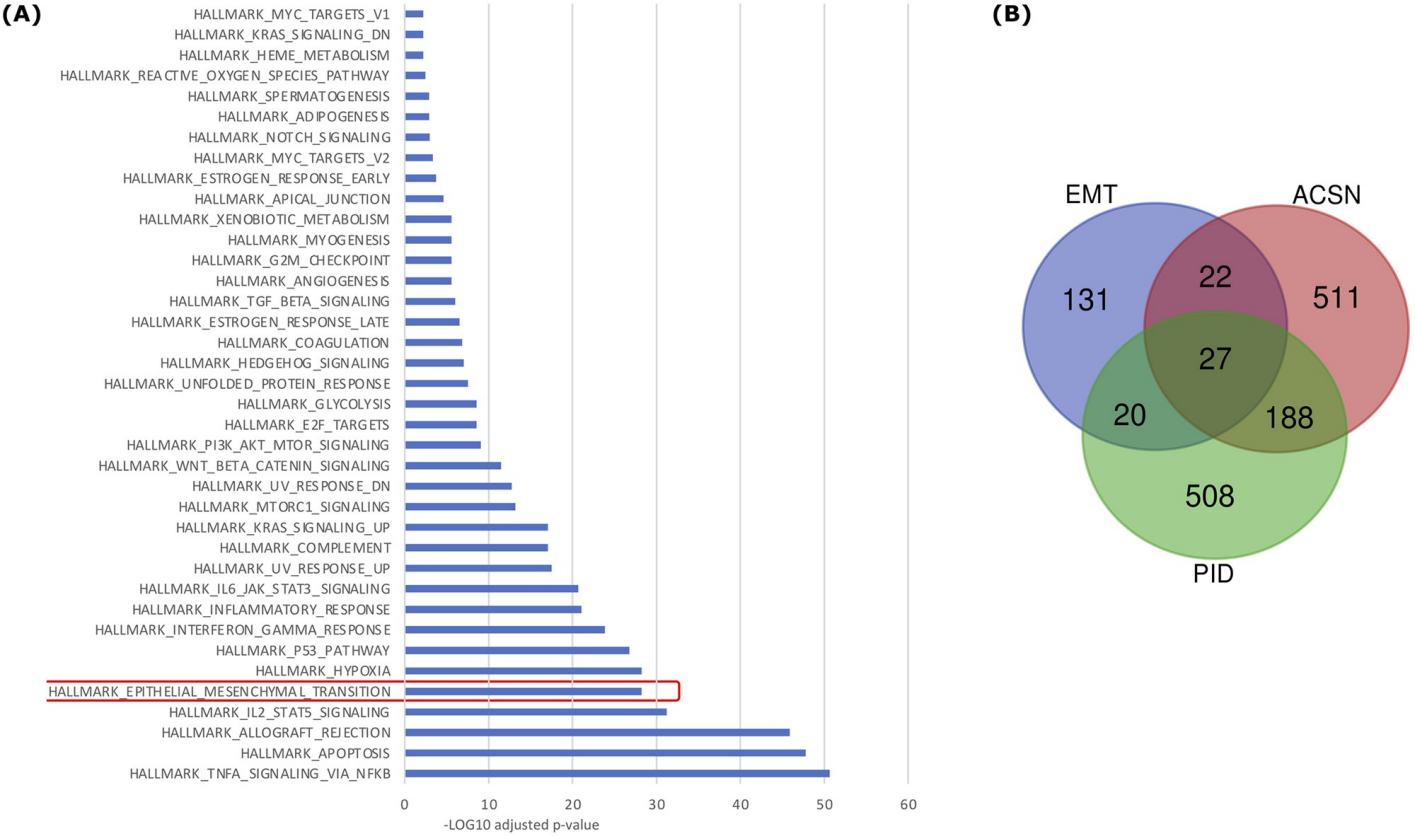
The PID and ACSN models are enriched in EMT genes. **(A)** Gene set enrichment analysis (GSEA) of the 215 genes (HUGO identifiers) common to PID and ACSN models. **(B)** Venn diagram describing the intersection between genes in PID and ACSN Cadbiom models and the EMT gene set from the MSigDB collection.

Together our results confirm a high degree of similarity between PID and ACSN models and to further explore their functional similarities, we searched for specific signatures of their 215 common genes. We performed gene set enrichment analyses using MSigDB collections from the Broad Institute (https://www.gsea-msigdb.org/gsea/msigdb/annotate.jsp) and among the 38 hallmark gene sets, we observed that the Epithelial Mesenchymal Transition (EMT) is one of the most significantly enriched gene sets (Fig 5A). Signaling pathways including TNF-alpha and IL2-STAT as well as apoptosis and allograft rejection gene sets were also enriched. Because EMT is a biological process including numerous signaling pathways, we decided to select the 27 EMT signature genes common to the PID and ACSN models (Fig 5B) to compare their controllers using the Cadbiom tool (see next paragraph).

### Analyzing controllers in the PID and ACSN models

The Cadbiom models can be dynamically analyzed using a non-deterministic framework for analyzing models in guarded transitions. The framework implements a partially parallel update, i.e., neither asynchronous nor synchronous, of the dynamics according to the implicit constraints carried by the guarded conditions and events. This semantics is an extension of Petri nets that is sufficiently expressive to account for all the biological mechanisms contained in the BioPAX model and transcribed in the Cadbiom model, such as complex associations, transcriptional regulatory mechanisms, transport or post-translational modifications. As this semantics is not deterministic, it cannot be used to simulate trajectories. However, with a reverse engineering approach, it is used to identify groups of molecules that are possible controllers of an expected phenotype.

As previously described [6], a phenotype is modeled by a state of the system, called *query*, described by a logical formula involving biological entities of the Cadbiom model. The *controllers* of the phenotype are all boundary entities of the model which are activated in at least one dynamical simulation of the model leading to the activation of the phenotype according to the guarded transition semantics. The *Trajectory entities* of the phenotype are all intermediate entities activated in at least one trajectory (see Materials and methods for details).

#### Causality analyses of phenotypes in the PID and ACSN models highlight the complementarity of the two databases

**Fig 6:**
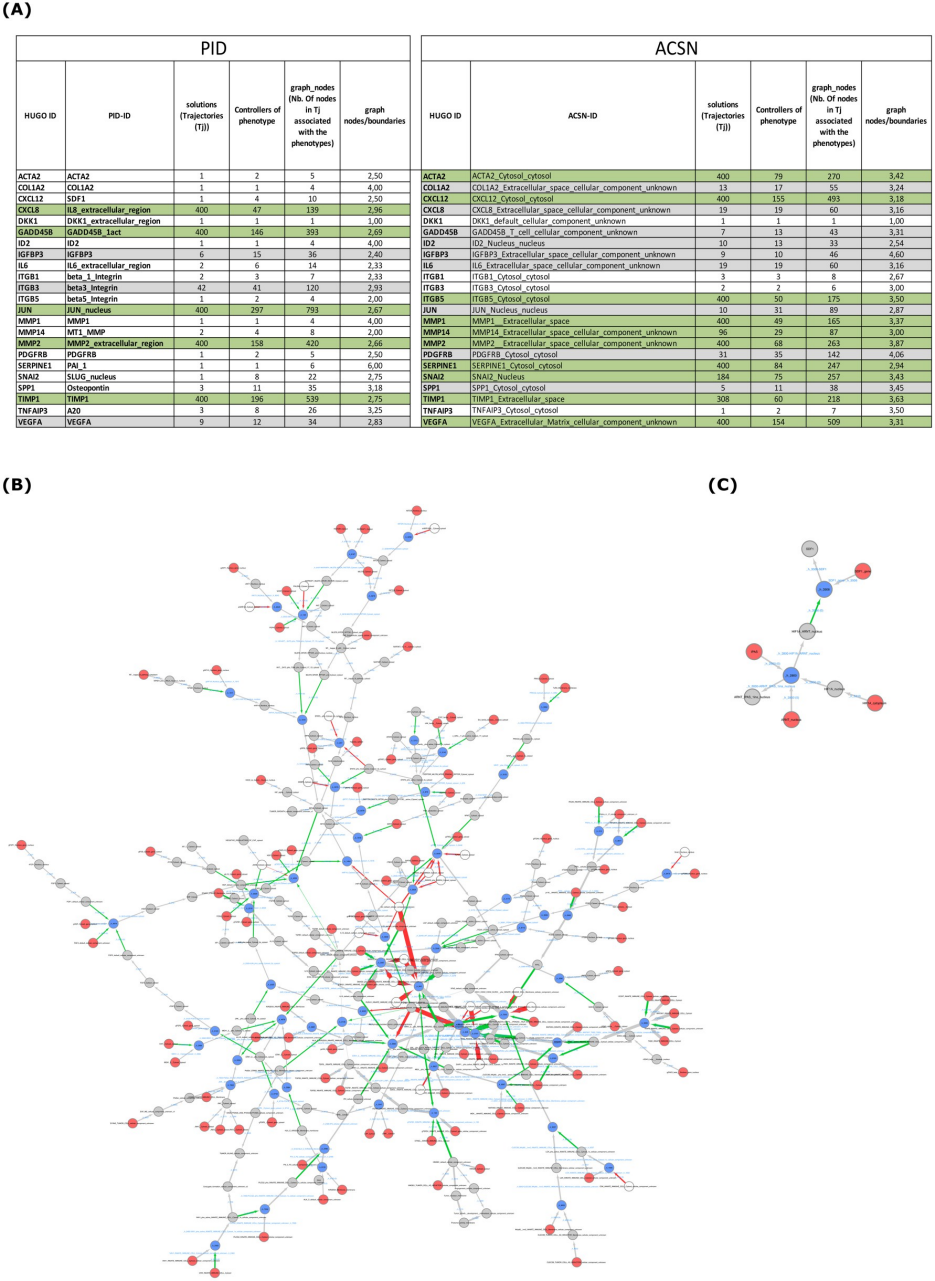
Comparison of EMT gene trajectories in the PID and ACSN models. **(A)** Analysis of 23 gene queries (phenotypes) in the PID and ACSN Cadbiom models. The first and second columns correspond to the HUGO and database identifiers, respectively. The third column describes the number of trajectories leading to the phenotype. The fourth column describes the number of controllers. The fifth column describes the number of nodes in all trajectories leading to the phenotype. The sixth column describes the ratio of the number of nodes in all trajectories to the number of controllers. Rows highlighted in green correspond to phenotypes with a high number of trajectories (from 96 to 400), grey rows correspond to phenotypes with an intermediate number of trajectories (from 6 to 42) and uncolored rows correspond to phenotypes with less than 3 trajectories. **(B and C)** Comparison of the trajectories to activate the CXCL12 gene in the ACSN **(B)** and PID **(C)** Cadbiom models. Graphical representations of trajectories. Red nodes are cadbiom model boundaries of the model. Grey nodes are basic entities/intermediate molecules which are not at the periphery of the model. Blue nodes denote reaction in which there are more than one reagent or one reactant (many-to-many or one-to-many relationships between reactants). White nodes are inhibitors, they are never in the solutions nor in the trajectories; their presence rule out the production/activation of molecules of interest. Grey arrows are unary reactions (one-to-one relationship). Red arrows are inhibitions and green arrows are activations (control reactions).

We used the mapping of the Cadbiom package to manually extract the Cadbiom identifiers from each of the HUGO gene identifiers for the 27 EMT genes shared by ACSN and PID databases. When several entities in the database could be mapped to a HUGO identifier, we considered all these entities. A Boolean formula for each of these identifiers was constructed. We performed *causality searches* to explore the dynamics of the PID and ACSN Cadbiom models for each of the Boolean formula by using the cadbiom-cmd package. It is important to note that each model has its own node description and manual curation is required to annotate them with HUGO identifiers. Therefore, automatic merging BioPAX files from the PID and ACSN databases is not possible. Fig 6A shows the results of the Cadbiom analysis for the 23 genes for which the search for controllers was successful. For each HUGO identifier, the corresponding biological entity in ACSN and PID with the highest number of controllers is indicated. Of note no trajectories were identified for 4 HUGO Identifiers either in PID (CXCL1) or ACSN models (FAS, WNT5A and SFRP1): this can be explained either by the absence of controllers or by the unreachability of the gene in our conditions.

We observed that the number of trajectories ranges from 1 to 400 (upper limit of the search) and the distribution is very heterogeneous across models. The genes of the PID model are associated with either a high number of trajectories (400 for CXCL8, GADD45B, JUN, MMP2 and TIMP1, green rows in Fig 6A), or an intermediate number of trajectories (42, 9 and 6 for ITGB3, VEGFA and IGFBP3, respectively, grey rows). The remaining 15 genes are associated with fewer than 3 trajectories (uncolored rows). In contrast, the EMT genes in the ACSN model are globally associated with more trajectories (400 for ACTA2, CXCL12, ITGB5, MMP1, MMP2, SERPINE1 and VEGFA, 308, 114 and 96 for TIMP1, SNAI2 and MMP14, respectively, green rows in Fig 6A). The remaining genes are associated with a wide range of trajectory numbers ranging from 1 to 31 (grey and uncolored rows) and only 4 genes have less than 3 trajectories (uncolored rows). This is consistent with the fact that the ACSN (Atlas of Cancer Signalling Network) database focuses on pathways involved in cancer where the epithelio-mesenchymal transition plays a critical role and is represented by a special map [21]. Consistent with this, the EMT genes of our panel are likely to be more documented in this database than in the generic PID database. To further illustrate this, we compared the trajectories of CXCL12, a key regulator of EMT and tumor invasion [24] in the PID and ACSN Cadbiom models. As shown in Fig 6A, we identified 155 and 4 controllers in the ACSN and PID Cadbiom models, respectively, and the comparative analysis of trajectory graphs illustrates the greater information in the ACSN model compared to the PID model (Fig 6(B) and 6(C)). It is important to note that the Cadbiom models cannot be used to rank genes based on the number of trajectories and controllers.

#### The combinatory of controllers in trajectories illustrates different complex regulatory networks in the PID and ACSN models

To further analyze gene controllers, we investigated the composition of the trajectories by extracting the number of boundary entities and the number of nodes in trajectories. We observed that the genes associated with a large number of trajectories (green rows in Fig 6A, greater than 40 trajectories) are also the genes for which the number of controllers is greater than 40 (except for MMP14 in the ACSN database). For genes with few trajectories, the association between the number of controllers and the number of trajectories was more variable: for instance, the SNAI2 gene has only one trajectory that contains 8 boundary entities in the PID model.

We calculated the ratio between boundary entities and the number of controllers to investigate the role of trajectory entities. We observed that the variation of this ratio is similar between the two models, from 1 to 6 (2.88 ± 0.96) in the PID model and from 1 to 4.6 (3.23 ± 0.66) in the ACSN model. Despite this similar ratio, we noticed that the composition of trajectories for a same gene can differ considerably between the two models. To illustrate this, we detailed the trajectories to activate the osteopontin gene (SPP1) in the two models. As shown in Fig 4 in S1 Appendix, there are 3 trajectories allowing SPP1 gene activation in the PID model, corresponding to 11 total boundary nodes that include signaling receptors (Syndecan_1,p75_NTR_1N_integral_to_membrane), ligand-receptor signaling complexes (FGFR_FGF, FGRF4_FGF19), receptor-interacting factors (NRIF,TRAF6), a signaling molecule (proBDNF_dimer_extracellular_region), a transcriptional regulatory complex (MDM2_KAP1_nuccleus), a transcriptional regulators (p53_1a_nucleus), a sorting receptor (sortilin) and a target gene (v2 Osteopontin_gene). In contrast, the trajectories for the SPP1 gene have different properties in the ACSN model. The 10 boundary entities of the 5 trajectories allowing SPP1 gene activation are signaling molecules (gPDGF_Cytosol_gene_cytosol, gSPP1_Cytosol_gene_cytosol, TGFB3_default_cellular_component_unknown, TGFB2_default_cellular_component_unknown, gTGFB1_Cytosol_gene_cytosol), signaling molecules from immune cells (TGFB3_INNATE_IMMUNE_CELL_Cytosol_cellular_component_unknown, TGFB2_INNATE_IMMUNE_CELL_Cytosol_cellular_component_unknown), a signaling receptor (gPDGFR_Cytosol_gene_cytosol), a scavenger receptor (STAB2_INNATE_IMMUNE_CELL_Membrane), a transcriptional regulator (JUND_Cytosol_cytosol), a cell phenotype (DYING_TUMOR_CELL_cellular_component_unknown) and a target gene (gSPP1_Cytosol_gene_cytosol).

This example illustrates the combinatories of controllers which can be either signaling molecules or transcriptional regulators but also complexes associating a growth factor with its receptor or two transcriptional regulators. The more information the database contains about a biological mechanism, the more controllers there are in the trajectories; and the more controllers are shared by different mechanisms, the more combinatories they are. To illustrate this, the contribution of the growth factors PDGF and TGF*β* in the regulation of SPP1 in the ACSN model was described by three trajectories differing only in the TGF*β* family members that include TGF*β*1, TGF*β*2 and TGF*β*3 (S1 Appendix).

### Combinatorics of controllers for EMT genes in the PID model

To better understand the richness of the combinatorial elements that make up the trajectories, we chose to detail a comprehensive analysis using two genes that illustrate the dynamics of the epithelial-mesenchymal transition process.

The epithelial-mesenchymal transition (EMT) is a critical step in tumor aggressiveness during which epithelial cells lose their cellular polarity and become mesenchymal cells with migratory and invasive properties. This complex process is induced by important changes in gene transcription and is characterized by numerous intermediate states with high cellular plasticity [25]. Changes in gene expression profiles during EMT have been widely documented [26], however dynamics of regulatory networks governing cell phenotypes remain unclear. In this context, we analyzed trajectories of gene expression regulation, specific to epithelial or mesenchymal states, using large-scale networks. According to [27], the epithelial phenotype is characterized by the expression of PERP which is an essential component of desmosome junctions and has been shown to preserve epithelial integrity. Similarily, according to [28], the mesenchymal phenotype is characterized by the expression MMP2 which is a metalloproteinase mainly expressed by mesenchymal cells that promotes cell invasion.

However, we noticed that the ACSN model does not contain the PERP gene, which is consistent with the fact that the ACSN database is focused on cancer pathways. Therefore, we chose the PID model to characterize the regulatory mechanisms of genes associated with epithelial and mesenchymal phenotypes. We used the causality search module to find the trajectories to activate either PERP alone, or MMP2 alone or MMP2 and PERP simultaneously in the Cadbiom model built from the PID database. In order to limit the computing time, we focused the analysis on the first 400 trajectories and evaluated the composition of these trajectories. All biomolecules present in all trajectories were analyzed using a clustering approach and heat-maps allowed us to easily compare the composition of the trajectories. In addition we used a graphical representation of all trajectories where nodes are biomolecules or reactions when there are more than one reagent or one reactant.

#### The Cadbiom signatures of the epithelial marker PERP and the mesenchymal marker MMP2 are specific

**Fig 7:**
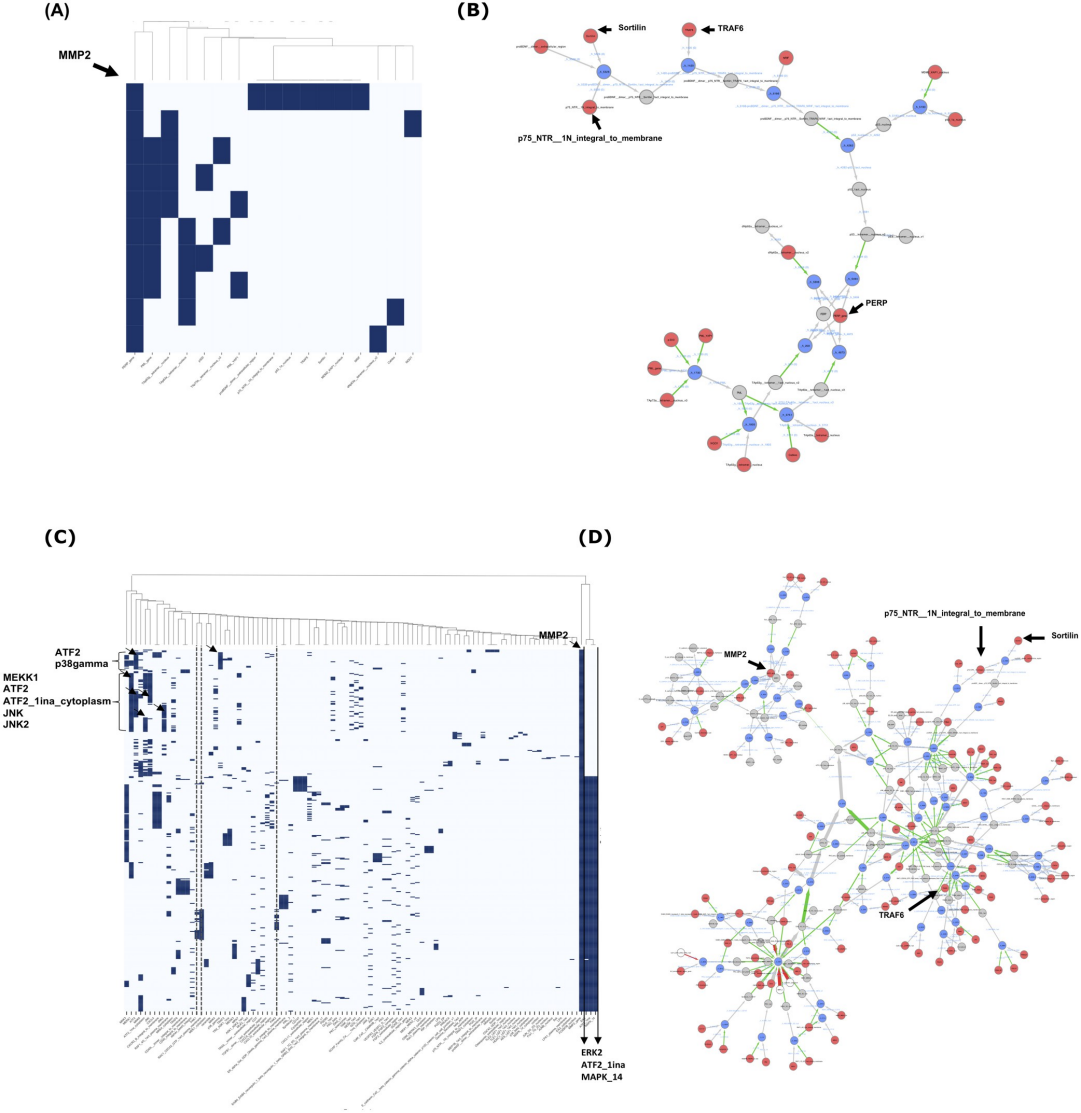
Analysis of trajectories obtained from PERP and MMP2 independent queries. **(A) & (C)**: Clustering analysis of trajectories based on controllers. PERP query (A) returns 10 trajectories including 17 controllers and MMP2 query (B) returns 400 trajectories including 101 controllers.**(B) & (D)**: Graphical representation of the trajectories resulting from PERP (B) and MMP2 (D) queries. Red nodes are the cadbiom model boundaries. Grey nodes are the basic entities/intermediate molecules that are not at the boundary model. The blue nodes are reaction nodes that are only displayed when there is more than one reagent or one reactant in a reaction (many-to-many or one-to-many relationship between reactants). The white nodes are the inhibitors, they are never in the solutions nor in the trajectories. Their presence is forbidden for the production/activation of the molecules of interest. The grey arrows are the reactions (unary reactions) (one-to-one relationship). The red arrows are inhibitions and the green arrows are activations (reaction controls).

The PERP query returned 10 trajectories that contained 17 controllers. Fig 7A shows the relationships between the trajectories (rows) and the controllers (columns). Fig 7B shows the molecules contained in the 10 trajectories: there are 12 intermediate compounds (grey nodes) that link the 17 controllers (red nodes) to the targeted PERP gene. The controllers include three members of the p53 family of transcription factors that are p53, p63 and p73. One of the trajectory illustrates the p53-dependent regulation of PERP expression [29]. It contains six controllers contributing to the activation of p53, including the MDM2-KAP1 complex that inhibits the acetylated form of p53, p53–1a and the p75-NTR-dependent activation of p53 along with its cofactor proBDNF, TRAF6, sortilin and NRIF [30]. In addition, six of the ten trajectories contain the transcriptional factor PML (Promyelocytic leukemia protein) associated with either TAp63a or TAp63g. TAp63 is one of the two isoforms of p63, which is characterized by the N-terminal transactivation domain (TA) in the promoter; TAp63a and TAp63g being isotypes with different C-terminal part of the protein [31]. These combinations may also include either the transcriptional co-activator protein p300, the transcriptional factor TAp73a, or the complex PML-YAP1 combining PML with the transcriptional regulator YAP1. In addition to the PML-dependent trajectories, two other trajectories contain either TAp63g combined with Quinone Reductase 1 (NQO1) or TAp63a combined with Cyclin-dependent kinase binding protein (CABLES). Note that one trajectory contains a single component, the dNp63a isoform that laks the TA domain and is known to be required for epithelium development and PERP regulation [32].

Compared with the regulation of the PERP gene, the trajectories related to the regulation of MMP2 are more complex with 101 components distributed over 400 trajectories (see Fig 7C), which are aggregated in the graph shown in Fig 7D. A remarkable observation is the presence of three components in 260 trajectories (65%). They are MAPK14 (p38*α* mitogen-activated protein kinase) and ERK2 (extracellular signal-regulated kinases) which are members of the MAP kinase family, and the inactive form of the transcriptional factor ATF2 (ATF2_1i in the model). Importantly, 86 additional trajectories (21%) are characterized by a similar signature including either p38gamma (22 trajectories) or JNK (64 trajectories) associated with inactivated forms of ATF2, i.e., ATF2–1ina-cytoplasm. Consistent with these observations, JNK (Jun N-terminal kinase), p38gamma (MAPK12), MAPK14 and ERK2 have previously been shown to phosphorylate the inactive form of ATF2 leading to its activation required for regulation of gene expression [33, 34]. These trajectories not only confirm the ATF2-dependent regulation of MMP2 [35, 36] but also provide new information upon the combination of kinases that were not included in the model. In support of this, we observed that the presence of JNK, p38gamma and MAPK14/ERK2 were mutually exclusive for the design of three types of regulatory pathways. As shown in the heatmap (Fig 7C), many controllers are not included in the 86 trajectories characterized by the presence of JNK or p38gamma suggesting specific regulation. These include PAK1 and PAK2, and the complex RAC1-CDC42-GTP (black dotted line) that exemplify the signalling pathway known to promote PAK-dependent EMT [37] and that has recently been implicated in the regulation of MMP2 [38]. Of note, the involvement of PAK1-dependent signalling in the regulation of MMP2 was not described in the PID database but revealed by the PID Cadbiom model. Together, these results show that our model not only clarifies the multiple trajectories involved in gene regulation, but also identifies new synergies and exclusions between pathways.

#### Combining queries for epithelial and mesenchymal genes characterizing an intermediate EMT phenotype

**Fig 8:**
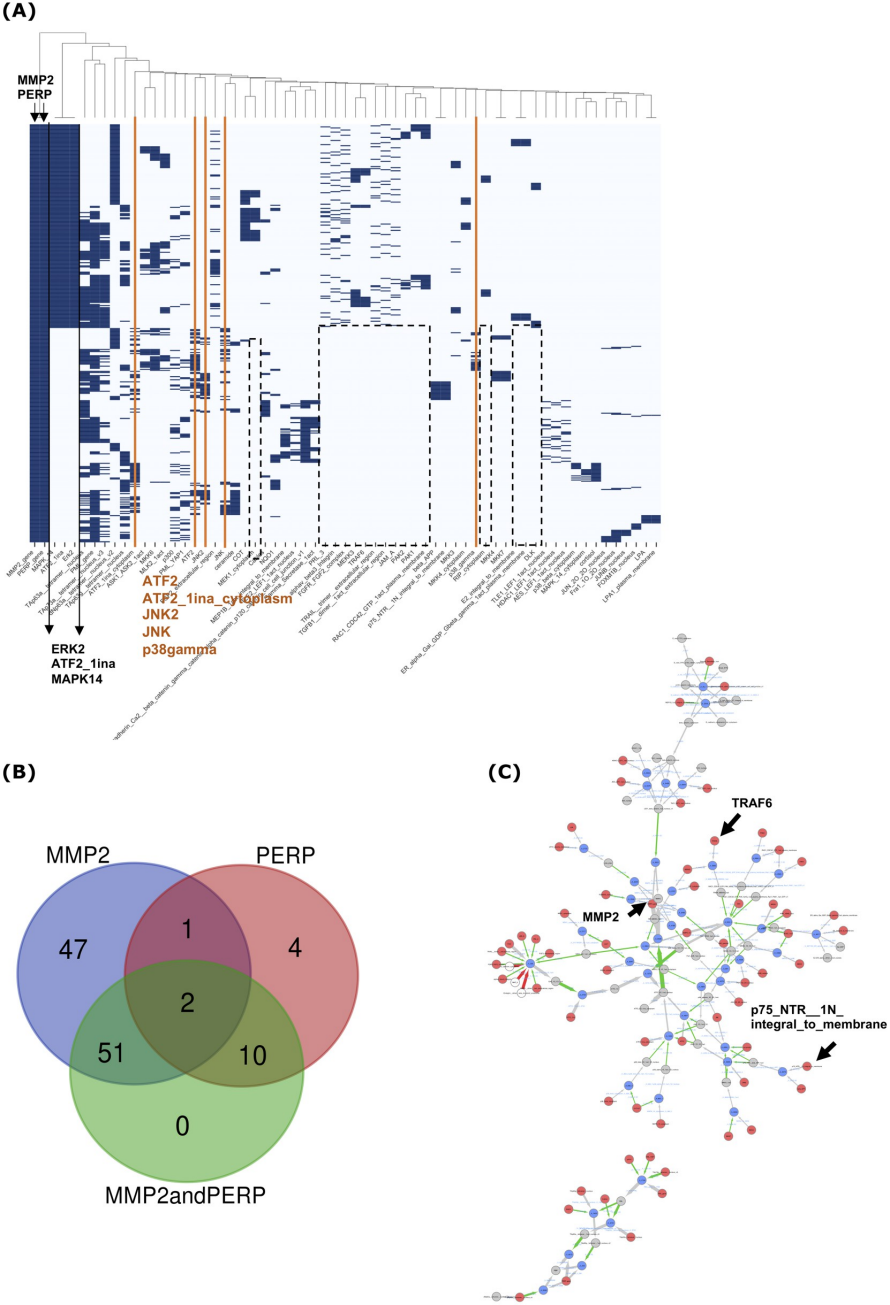
Analysis of trajectories obtained from the “PERPandMMP2” query. **(A)** Clustering analysis of trajectories based on controllers (400 trajectories and 98 controllers). **(B)** Venn diagram describing the intersection between controllers obtained from MMP2, PERP and “PERP and MMP2” queries. **(C)** Graphical representation of the 400 trajectories.

A major feature of our framework is the ability to combine queries to study complex phenotypes. To explore the dynamic changes during EMT, we searched for regulatory pathways that explain both the expression of PERP and MMP2, that respectively characterize epithelial and mesenchymal cells, that could characterize an intermediate state of EMT. Analysis of the 400 trajectories for the “PERPandMMP2” query (rows of Fig 8A) identified 63 controllers (columns of Fig 8A), of which 10 and 51 are found in the PERP and MMP2 specific queries, respectively (see Venn diagram in Fig 8B). Trajectories are aggregated in the graph shown in Fig 8C. It is important to note that no new controllers were generated by this combined query and that 47 controllers of the MMP2 trajectories were lost in the results of the “PERPandMMP2” query. Most of these are associated with cell responses to the extracellular microenvironment. They include inflammatory response players such as CX3CR1 and its ligands, CXCL9, CXCL10, CXCL11 and CXCL4 [39], CD40 and its ligand CD40L [40], and IL2 [41]. Similarly agents that modulate cell proliferation and migration such as growth factor receptors including ERBB4 and VEGFR3, and the secreted cytokine OSM (Oncostatin M) [42] are lost in the “PERPandMMP2” query compared with the MMP2 query alone. We also observed the loss of cell surface receptors that mediate the interaction between cells and the extracellular matrix such as the proteoglycan syndecan 1 which plays a central role in cell adhesion and migration [43], CD147 which is known to induce MMP2 expression in fibroblasts [44], and osteopontin which induces MMP2 and cell invasion [45]. Similarly to the loss of controllers in MMP2 trajectories, 4 controllers from PERP trajectories were absent in the trajectories of the “PERPandMMP2” query. They are p53_1a_nucleus, proBDNF__dimer__extracellular_region, NRIF and MDM2_KAP1_nucleus which are associated with the p53-mediated cell death pathway [30]. The other two actors in this pathway, p75NTR and TRAF6, are both present in the MMP2, PERP and “PERPandMMP2” queries highlightening the p53-independent implication of p75-NTR and TRAF6 in MMP2 trajectories. Indeed, looking at the MMP2 graph (Fig 7D), we observed that p75-NTR is associated with APP (Amyloid protein precursor) and NGF-dependent paths which is in agreement with a previous report on the interaction between p75-NTR and APP, and the modulation by nerve growth factor [46]. Similarly TRAF6 is associated with the CD40 signalling pathway [47] in MMP2 trajectories while TRAF6 is associated with the p53-mediated cell death pathway in PERP trajectories. In addition, sortilin which is a well-known cofactor of nerve growth factor dependent signalling [48] is lost from “PERPandMMP2” trajectories while present in MMP2 and PERP trajectories. This observation suggest that sortilin is differentially involved in PERP and MMP2 trajectories. Finally, 10 of the 14 controllers specific to PERP trajectories are conserved in the combined “PERPandMMP2” query suggesting that they are independent from MMP2-dependent trajectories. Together, our results suggest that the combination of PERP and MMP2 queries introduce new constraints on the model, which mainly reduce the ways to activate MMP2 and illustrate an intermediate stage of EMT.

## Discussion and conclusion

The understanding of molecular mechanisms underlying complex biological processes relies today on the use of databases and computational methods for large-scale networks. The diversity of both languages used to formalize biological events, and tools used to model them, confronts the biologist with choices that must take into account the type of data and the question asked. Considerable efforts have been made to standardize formalisms and BioPAX is now a widely used language for representing biological pathways [7]. Because of its interoperability, the BioPAX language can facilitate the integration of data from different sources. However, to our knowledge, there is only one tool for simulating biological pathway models specified in BioPAX, BioASF, a framework based on the principles of discrete event systems and multi agent systems [17]. Although this tool can simulate the models described in BioPAX, the need to manually define its own simulation rules for each model limits its use to models that are already finely described and of very small size.

For the first time, we developed a framework that enables the dynamical modeling of large-scale models, formalized in the BioPAX language. A main contribution of this work is the redesign of an earlier version of the Cadbiom tool [6] which includes a module to convert a database described in the BioPAX format into a dynamical model based on guarded transitions, a module to explore and to identify the trajectories and controllers with respect to an expected phenotype (e.g., gene activation) and a module to facilitate visualizing analyses by graphs and heatmaps. Applied to the PID and KEGG BioPAX files from the Pathway Commons database and to the curated ACSN BioPAX file, we compared the structures of the resulting models and demonstrated their high level of complementarity. Finally, our case-study highlighted the added value of Cadbiom in deciphering the combined effect of controllers on genes activation.

### High level of complementarity in the BioPAX content

Investigating the structure and the content of the BioPAX files reveals disparities between the PID, KEGG (extracted from the Pathway Commons database) and ACSN files. The differences in the type and number of BioPAX classes highlight that KEGG is a metabolism database with metabolic reactions and an abundance of small molecules. It should be noted that the original KEGG database encompasses not only metabolism pathways but also many other biological pathways [49] that do not appear to be available as BioPAX files in the Pathway Commons database. On the other hand, the PID and ACSN models share many features including the number of transitions and boundary entities. Finally, the analysis of the controllers allowed us to discriminate between the PID and ACSN models, revealing different dynamics. Comparison of the models raises the question of the best description of the biological information according to the BioPAX classes and demonstrates the complementarity of the Cadbiom models. Applied to the PID, KEGG and ACSN databases, our approach has thus allowed to extract and curate the relevant information from the BioPAX description of the databases, in order to interpret them in a unified dynamical model despite their initial heterogeneity.

### Towards the combined analysis of BioPAX databases

Although the three BioPAX databases we analyzed are very complementary, several limitations prevented us from merging the databases to take full advantage of their complementarities. The main limitation is related to the lack of standardization in the naming of several entities, such as complexes that do not follow a unified framework for their identifiers. Special attention should be paid to the fact that BioPAX models are often incomplete. A molecule can be associated/transformed into different complexes with different locations, which prevents automatic model merging. Our strategy has been to work with unified graphs for each model rather than fusioning models.

The most promising approach to combine BioPAX models into a single Cadbiom model is to adopt a strategy similar to the method implemented for the construction of the OmniPath database [15] which aggregates multiple databases into a unified interaction graph of signaling pathways. In this paper, the authors manually selected the contents of the databases corresponding to standardized entities and focused on a limited but well-curated number of reactions and classes of biological entities. The result is large-scale interaction graphs, which, however cannot be analyzed with dynamical methods: as shown in [16], this resource seems to be of great interest to manually build custom Boolean models, but requires an important manual curation procedure to interpret the OmniPath interaction graph into a Boolean model. Our plan is to adopt a strategy similar to [15] to extract information from multiple BioPAX resources on a subset of typed entities. This will provide us with a unified data source that will automatically be then interpreted in a dynamical Cadbiom multi-source model.

### Using guarded-transition models to decipher multi-scale models

The originality of the Cadbiom approach is that the controllers are computed to take into account all the competitions and control processes in the molecular transformation chains leading to the targeted entities. Indeed, our approach relies on guarded transitions, a logic-based formalism that is an extension of Petri nets to model complex control events [6]. The choice of this formalism was motivated by the objective of identifying controllers of large-scale molecular interaction networks involving both transformations and their controls. More precisely, modeling the dual role of transformations (such as metabolic reactions or complex formation) and controls (such as regulation of signaling reactions) has always been a main cornerstone in systems biology, as these two classes of reactions have different time scales and represent different types of transformation. This duality is found in the BioPAX ontology since transformations and controls correspond to different classes (conversion classes vs control classes). We demonstrate here that, following the framwork introduced in [6], the Cadbiom models, by modeling the transformation of biological entities with guarded transitions, encompass all multi-scale biological transformations in a single framework in agreement with the BioPAX ontology. Indeed, Cadbiom transitions are appropriate to model biological transformations (such as metabolic reactions or complex formation) while the Cadbiom guards are appropriate to transcribe control mechanisms such as signaling regulation. By integrating transformation and control reactions into a single formalism, it becomes possible to analyze causalities in the entire netwok by solving SAT-encoded satisfaction formulas. The transformation rules between the BioPAX ontology and the Cadbiom models are thus a key feature for the identification of regulated transformation chains and the computation of phenotype controllers.

### The epithelio-mesenchymal transition as a paradigm of the complexity of regulatory pathways

EMT is orchestrated by numerous changes in regulatory pathways [19] and understanding how these molecular pathways act together requires an integrative and dynamical view that considers combination, competition and control events in large-scale models. Using the Cadbiom model built from PID knowledge encoded in the BioPAX format, we identified a large number of controllers for the EMT markers, PERP and MMP2. Importantly, the predicted controllers in the trajectories for PERP and MMP2 were supported by the published literature (21 Pubmed references) and only 3 references were initially present in the PID database that contains 4890 references. This demonstrates the predictive value of the Cadbiom models. Moreover, the simple query for either an epithelial (PERP) or mesenchymal (MMP2) marker returned separate controller signatures whereas a combined query associating PERPandMMP2 did not lead to the sum of each query but generated a new signature characterizing an intermediate cellular state. This is consistent with the fact that EMT is not a linear process and that cell phenotypes evolve during EMT leading to “multiple or hybrid transition phases” as defined in [50]. Of course high-throughput approaches including transcriptomic, proteomic and epigenomic analyses have been developed to capture such heterogeneity but the integration and modeling of these results remains a challenge [51]. Taking advantages of the BioPAX language and using a discrete modeling approach based on guarded transitions, we developed a novel framework for exploring the combination of regulatory pathways in large-scale networks. Future research will aim to develop more complex queries to explore a range of changes in controller signatures to identify key regulators for therapeutic targeting.

### Applying Cadbiom to other BioPAX databases

The current release of the Pathway Commons database (Version 12) contains 24 databases that all have a BioPAX file that could be converted in a Cadbiom model with a similar approach. However, this requires a manual analysis of the internal structure of each database to identify the keys for conversion to a Cadbiom model. In this case, a special attention must be paid to the use of nested classes to represent biological processes. These classes are described by generic entities that themselves contain other generic entities and require specific curation procedures in order to interpret the model dynamically. In the models we considered, these nested classes and the underlying generic entities could be totally expanded in a two-step procedure. By contrast, we observed that, the Reactome pathway database [52] contains 13.5% nested entity classes among its 42,349 physical entities, so their expansion cannot be achieved with a naive strategy. Based on the present work, our future plan is to apply the Cadbiom framework to the Comparative Toxicogenomics Database (CDT) [53] which provides a very broad range of biological toxicology information including chemicals, genes, proteins, phenotypes, pathways, diseases, and their relationships. The BioPAX CTD model is available on the Pathway Commons website and the interpretation of the BioPAX CTD model in the CTD Cadbiom model will allow us to explore how environmental exposures affect health.

## Supporting information

S1 Appendix This section contains supplementary Material and Methods including six subsections: 1) Curation of PID, KEGG and ACSN BioPax models; 2) Rewriting strategy of BioPAX models into Cadbiom models; 3) Comparison of Cadbiom models; 4) Dynamics of guarded-transition models; 5) The Cadbiom framework and 6) Trajectories analysis in the PID and ACSN Cadbiom models.
(PDF)

S1 Table List of genes in the PID and ACSN models (HUGO gene symbols). (PDF)
