# Supplementary material for: Discrete modeling for integration and analysis of large-scale signaling networks
Source: PLoS Comput Biol. 2022 Jun 13;18(6):e1010175. doi: 10.1371/journal.pcbi.1010175 (PMC9232147; doi:10.1371/journal.pcbi.1010175)
Supplement: S1 Appendix — This section contains supplementary Material and Methods including six subsections: 1) Curation of PID, KEGG and ACSN BioPax models; 2) Rewriting strategy of BioPAX models into Cadbiom models; 3) Comparison of Cadbiom models; 4) Dynamics of guarded-transition models; 5) The Cadbiom framework and 6) Trajectories analysis in the PID and ACSN Cadbiom models. (PDF) [file pcbi.1010175.s001.pdf]

# Discrete modeling for integration and analysis of large-scale signaling networks

S1 Appendix

## 1 Curation of the PID, KEGG and ACSN BioPax models

**Homogenization of ACSN with respect to the other databases** We noticed that the BioPAX file describing the ACSN database frequently instantiates the generic types of the ontology (`Entity`). Only 11,922 entities have the types `Proteins`, `Complexes` and `SmallMolecules` among the 27,426 entities according to the names of the entities described in the BioPAX file, the other entities being generic. To facilitate comparison with the other databases, we limited the analysis of ACSN database to these entity types and their associated interactions. The characteristics of the databases, including the curated ACSN database, are presented in Table 1.

**Curation of PID with respect to generic entities, nested classes and duplicated entities** We noticed that the PID can sometimes group collections of molecules in generic BioPAX entities, using the `MemberPhysicalEntity` property. For example, all molecules involved in a common biological process such as a change in cell compartment, can be grouped in such generic entities. This facilitates the representation of biological phenomena in visualization software such as Chibe, or on web-based platforms such as Pathway Commons. However, nearly 30% of the generic entity classes in PID do not appear in the reactions of the database, suggesting that the models could be reduced without loss of information about their dynamical properties.

We also observed that the PID database uses nested classes to represent biological processes. These classes are described by generic entities that themselves contain other generic entities. As shown in Table 1, the PID contains 5.7% (23/403) of nested entity classes, which leads to a complex hierarchical structure requiring specific curation procedures in order to interpret the model dynamically. 157 of the generic entity classes of the PID were annotated with the characteristic property of the PhysicalEntity class named `ModificationFeature`. This property is important because it describes post-translational modifications characterized by the addition of atoms or groups via covalent bonds (as in adenylation and phosphorylation processes).

In order to homogenize and curate the databases, we removed classes (generic entities) and nested classes when they were not involved in BioPAX reactions or controllers. When this removal was implemented, the member entities inherited the properties `ModificationFeatures` and `Location` from their parent class.

The analysis of `PhysicalEntities` in the databases highlighted that several entities could be considered *duplicated*, as they shared the following properties: type, entityReference, displayName, cellularLocation, feature (ModificationFeature only). The entities actually differed with regard to information unrelated to the reactions, such as different Pubmed references or the stoechiometry of the components of the complexes. All databases contained such duplicate entities (from 74 in the ACSN to 699 in the PID models). We processed the databases to group all duplicated entities together, so that each group of similar entities eventually appeared in the processed model as a single entity with a unique identifier.

**Curation and study of `Interaction` entities based on controls and reactions highlights that the KEGG BioPAX ressource contains little information on signaling pathways** We have denoted as *Reactions* all entities of the subclasses `Conversion` and `TemplateReaction` of the class `Interaction`. The type `TemplateReaction` (transcription or translation of DNA or RNA) is mainly used in the PID BioPAX file; it allows the identification of physical entities considered as genes.

In the BioPAX resources, `Controls` are mainly depicted by the types `Catalysis` and `TemplateReactionRegulation` (see Table 1). The controls of type `Modulation` are present at low frequency. Therefore they were not considered in our parsing procedure. Controls of type `Catalysis` for which the controlType property was absent (in the KEGG resource) were considered activation by default.

We noticed that all databases contain biomolecules playing both roles of product and reagent of the same `Interaction` (from 50 to 934 reactions depending on the database). We considered that they played the role of catalysts, and therefore removed them from the list of reactants to create new objects

Catalysis regulating the concerned reactions. This curation procedure resulted in an increase in the number of controls (post-translational changes) in the PID (6,195=6,145+50), KEGG (2,716=1,782+934) and ACSN (6,519=6,186+333) models.

As shown in Table 1, the PID and ACSN BioPAX databases contain few SmallMolecules and the reactants of reactions are mainly Proteins and Complexes. This type of content is representative of signaling pathways with proteins involved in either signalling reactions or control of these reactions.

In contrast, the KEGG BioPAX file contains proportionally more SmallMolecules (1,585) than the other databases but no ComplexAssembly reactions are found. There are as many BiochemicalReaction (1,786) as Control reactions (1,782) (only from the class Catalysis) suggesting that each reaction is effectively catalyzed by a single biomolecule. This is because the KEGG BioPAX file consists specifically of small molecules and corresponds to the metabolism part of the entire KEGG database, with no description of the signalling part.

# 2 Rewriting strategy of the BioPAX models into the Cadbiom models

Each physical entity described in a BioPAX file (protein, small molecule, RNA, DNA, Complex) has been associated with a Cadbiom biomolecule, characterized by a unique identifier. These identifiers are automatically constructed from standard entity names or generic identifiers (HGNC, Chebi) defined in the BioPAX data, according to internal grammatical rules in order to distinguish the state of a biomolecule (activated/inactivated, phosphorylated, ubiquitinated, etc.) and/or its location. Particular attention was paid to the identification of gene-related entities in the BioPAX resources. We noticed that none of the resource uses the BioPAX Gene class. Instead, they follow the BioPAX recommendation to use the class TemplateReaction to formalize transcription, translation and gene expression reactions (DNA to RNA, RNA to Protein, and DNA to Protein). However, in the resources, these interactions have products but they may not have input biomolecules. For example, PID has 1,492 template reactions associated with gene expression, but contains 0 instance of Dna and 22 instances of Rna. Other databases such as ACSN use the BiochemicalReaction class with types Dna and Rna as reagent and formalize gene expression using two successive BiochemicalReactions for transcription and translation. In Cadbiom, we define as *genes* all entities having already in their name the substring "_gene", or having the BioPAX Dna type, or participating in TemplateReaction. In all these cases, we have systematically created a new Cadbiom entity , whose identifier is the concatenation of the name of the product of the interaction and a "_gene" suffix.

The guarded transitions were automatically constructed from the BioPAX interactions (Biochemical-Reaction, ComplexAssembly, Transport, Degradation, Transport with biochemical reactions and TemplateReaction). As these interactions correspond to the transformation of reagents into products, the main rewriting principle described above naturally applies. An illustration of this case is given in Fig 2A. The control classes (Catalysis, TemplateReactionRegulation, Control and Modulation) were used to define the conditions necessary for the completion of the guarded transitions associated with the regulated reaction. These BioPAX Control subclasses were therefore used to construct logic formulas embedded in the condition of the corresponding guarded transition. We assumed that the presence of at least one activator and the absence of all inhibitors are required to activate a control. This is the most permissive rule in the absence of information - in the BioPAX files - about how controllers of the same reaction may interact together. An illustration of this case is given in Fig 2B. If the regulation is operated by a class of physical entities, we have assumed that any of the member entities is an independent relevant regulator.

The concept of class and the problem of duplicated entities, highlighted by the analysis of the content of the BioPAX models, require special attention. Indeed, the mapping to a dynamical model requires deleting all the classes and replacing them by all their corresponding entities. To avoid an explosion of the number of events in the model and reduce the final guarded transitions model, we interpreted BioPAX models involving classes of entities according to three main principles. *(a)* Duplicated entities are mapped to a single representative entity according to a method that allows tracing of the mapping. *(b)* Classes are expanded only if one of their components is involved in another reaction; in this case, the reaction is duplicated for all members of the class used in the BioPAX model, the other entities are ignored. On the contrary, if none of the entities of a class are in the BioPAX model, the class is not expanded and

is kept in the Cadbiom model (see example in Fig 2C). *(c)* Cellular localization and post-translational attributes associated with classes of biological entities are systematically propagated to all biological entities contained in the considered classes (see example in Fig 2D). These principles are used in more than 30 different sub-cases (involving classes in input, output or regulation conditions) which are described in the software documentation website http://cadbiom.genouest.org/doc/biopax2cadbiom/examples.html.

# 3 Comparison of the Cadbiom models

Although it has not been updated since 2014, PID stays one of the most documented pathway databases as demonstrated by the comparative analysis of 55 biological resources in [1]. In 2015, the authors of the ACSN project concluded that: "... the content of ACSN is not redundant with the other pathway databases compared, though the most canonical molecular pathways are represented similarly in all of them" [2]. The ACSN database is organized as biological process maps including Cell Cycle and DNA repair, Cell Survival, Regulated Cell Death, Telomere maintenance, EMT and Senescence, Invasion-Motility, Angiogenesis, Adaptive Immunity, Innate Immunity, Cancer-Associated Fibroblasts, Dendritic Cell, Natural Killer Cell and Macrophages-MDSC. Each map contains modules with pathways characterizing the maps. By contrast the PID database is organized as 77 signaling and regulatory pathways [3] such as growth factor signaling pathways (e.g., TGF-beta receptor signaling), cell receptor pathways (e.g., a6b1 and a6b4 Integrin signaling) and intracellular pathways (e.g., p38 MAPK signaling).

Comparing the PID and ACSN Cadbiom models, we identified PID "pathways" which are not documented in the ACSN model including: - The leptin signaling pathway; In the PID model, LEP (leptin) interacts with LEPR which control expression of SOCS3 and phosphorylation of STAT3. In the ACSN model, LEP and LEPR are absent - The IL27RA Signaling pathway; In the PID model, IL27RA (interleukin 27 receptor) interacts with IL27, EBI3, TYK2, IL6ST, JAK1, JAK2. It controls phosphorylation of STAT member family (STAT1 to 5) and controls expression of IL2 and INFG. In the ACSN model, ILR27RA and IL27 are absent - The IL23R signaling pathway; In the PID model, IL23R (interleukin 23 receptor) interacts with JAK2, SOCS3 and TYK2. It controls expression of ALOX12B, CCL2, CD3E, CD4, CXC1, CXCL9, IFNG, IL19, IL1B, IL24, IL6 ITGA3, MPO and controls phosphotylation of NKKB1, NFKB1A, PI3KCA, PI3KR1, RELA, TNF, STAT1 and STAT5. In the ACSN model, IL23R is absent but its binding partner IL12RB1 is present - The Neurotrophic factor mediated Trk signaling pathway ; In the PID model, NTKR1 (Neurotrophic Receptor Tyrosine Kinase 1) controls the phosphorylation of FRS2, DNAJA3, and GAB1. In the ACSN model, NTKR1 is absent. -The Circadian rhythm pathway; In the PID model CRY2 (Cryptochrome Circadian Regulator 2) controls expression of ARNTL and CLOCK and interacts with CSNKIE, PER1, PER2 and TIMELESS. In the ACSN model, CRY2 is absent.

On the other hand, specific processes are enriched in ACSN such as the senescence network which is not specifically documented in PID. It is difficult to compare an "ACSN module" with a PID pathway since the module gathers more information. For example the WNT canonical module in ACSN contains 200 proteins while the WNT signaling pathway in PID only contains 28 proteins.

**Boundary nodes can have two different roles** . The classification of boundary entities by type and by role played in transitions is detailed in Table 2 and illustrated in Fig 1 in S1 Appendix . They have two different roles. First, they can be the input of a guarded transition, thus corresponding to biomolecules that initiate a pathway modeled by a chain of guarded transitions. There are 3,324, 386 and 3,078 such biomolecules in the PID, KEGG and ACSN models, respectively (Table 2). In the case where the corresponding BioPAX reaction has more than two inputs, the boundary is also a member of the logical formula describing the condition of the guarded transition of the model. Second, boundary nodes that are members of a guarded condition but are not input of a transition play a completely different role: they correspond to regulators of reactions, such as biomolecules involved in Control BioPAX reactions. There are 699 (=2442-1743), 1032 and 615 such biomolecules in the PID, KEGG and ACSN models, respectively. Therefore, control boundary nodes represent 17.8% (=699/3925) of the total number of boundary nodes in the PID model, and 72.6% and 16.6% in the KEGG and ACSN models, respectively. This is consistent with the fact that PID and ACSN aggregate several independent maps, generating a large number of biomolecules initiating the corresponding pathways, e.g. input boundary entities. On

the contrary, KEGG is characterized by a large number of enzymes controlling reactions and rather few input boundary entities.

# 4 Dynamics of guarded-transition models

We use the formalism of guarded transition to analyze the dynamics of `Cadbiom` models according to a non-deterministic framework. The formalism is inspired by the UML state transition semantics enriched by an algebra: a guarded transition $t : A^{h[Cond]} \to B$ is activated as soon as its input $A$ is activated and its condition $Cond$ is satisfied. The time-clock $h$ allows synchronizing the activation of several transitions participating in the same event (in the case of complex reactions). In practice, thanks to the `Cadbiom` framework, each event and each entity is associated with Boolean variables. A system state is described by the values of these variables. In the implementation of `Cadbiom`, a state $s_1$ is defined as reachable from another state $s_0$ if there exists a set of guarded transitions $t_1, \ldots t_n$ that share the same event $h$ such that *(i)* each reagent of the selected guarded transitions is activated in the state $s_0$ and inactivated in the state $s_1$ (i.e. the transitions inactivate their inputs), *(ii)* each product of the selected guarded transitions are activated in the state $s_1$ (i.e.the transitions activate their outputs), *(iii)* all logical formulas describing conditions of the guarded transitions are satisfied in the state $s_0$.

According to this formalism, we highlight four concepts of nodes of interest with the analysis of the dynamics of a `Cadbiom` model. The first one is related to the complete `Cadbiom` model whereas the three other ones are related to phenotypes, which are interpreted as logical formula describing the activation or the inhibition status of the entities involved in the phenotype. It can be associated with a state $s$ of the `Cadbiom` model.

- *Boundary entities of the* `Cadbiom` *model* (named "frontier places" in the seminal paper [4]) are all entities of the full cadbiom model which are not the output of guarded transitions. By default, they are assumed to be activated at the initial state of a system simulation.
- *The controlers of a phenotype* are all boundary entities of the `Cadbiom` model which are activated in at least one dynamical simulation of the model leading to the activation of the phenotype according to the guarded transition semantics. The controlers of $s$ are all boundary entities of the `Cadbiom` model activated in at least a state $s0$ such that $s$ is reachable from $s0$.
- *The trajectory entities of a phenotype* are all intermediate entities of the `Cadbiom` model activated in at least one trajectory leading to the phenotype. More precisely, if $s$ is the state associated with the phenotype query and $s0$ is a initial state of a dynamical trajectory, the trajectory entities of $s$ associated with $s_0$ are all entities activated in at least one intermediate state along the succession of states $s_0, s_1 \ldots, s$ reaching $s$. The (aggregated) union of the trajectories associated with all boundary controllers of $s$ is called trajectory of $s$.
- *The* `Cadbiom` *signature of a phenotype* encompasses all controlers and trajectory entities of the phenotype according to the `Cadbiom` semantics.

# 5 The `Cadbiom` framework

The `Cadbiom` framework and its suite (GNU GPL License) is written in Python and hosted on the PyPI (Python Package Index) platform for easy deployment. The installation procedure, documentation for users and developers, sources and examples illustrating the interpretation processes of large-scale databases are detailed at `http://cadbiom.genouest.org`.

The `Cadbiom` framework is composed of four python packages described in Fig 2 in S1 Appendix: *cadbiom-gui* (Graphical User Interface) and `cadbiom-core` (library) are fully refactored packages from their former versions [4]. The packages `cadbiom-cmd` (command line) and the `biopax2cadbiom` (conversion of BioPAX models into the Cadbiom models) are designed specifically for the new version of `Cadbiom` used to produce the results in the present study.

The `cadbiom-cmd` package includes subcommands for working with input and output files of the `Cadbiom` suite, from a command line interface.

The `cadbiom-core` library itself depends on a SAT solver, the open source CryptoMinisat solver [5]. This solver is used to solve constraints modeling reachability problems in guarded transitions large-scale models for computing controllers. The SAT solver is used either to return a satisfiability answer, or to generate a set of phenotype controllers. In practice, the `cadbiom-cmd` package executes the `cadbiom-core` package in order to identify the families of biomolecules corresponding to the initial conditions of the system that led to the satisfiability of the Boolean formula. These biomolecules are the controllers of the targeted phenotypes of the model.

The `biopax2cadbiom` package is a stand-alone module, also integrated in the `Cadbiom` Graphical User Interface (GUI). biopax2cadbiom is a module that aims at creating cadbiom models from BioPAX resources. It relies mainly on SPARQL endpoint queries and thus uses completely different technologies than cadbiom. As the two packages use completely different technologies (and dependencies) with different objectives (creation of a cadbiom model versus analysis of a cadbiom model), the modules have been designed independently. `biopax2cadbiom` is compatible with the BioPAX Level 3 specifications, which offers the possibility to define entities under several states (including a generic state). The tool executes queries in the SPARQL query language to automatically create Python classes based on the BioPAX specifications. The objects instantiated from these classes are then used in the translation operations of the BioPAX formalism to the `Cadbiom` format.

The Graphical User Interface provides a graph editor to build and explore models, with a simple query interface to analyse the dynamics of a guarded-transition model.

In order to adapt to large-scale models analysis, the former library developed in [4] was modified as follows: (i) time-consuming functions were rewritten in C language, (ii) an architecture implementing multiprocessing technology was integrated into the command line module, (iii) the cryptominisat solver was upgraded. Overall, these changes increase the capabilities of `Cadbiom` such that the performance of the current implementation is 2000 times faster, for the same query on a single CPU, than the original implementation.

The `Cadbiom` framework is developed and used to compute boundary and trajectory controllers of phenotypes modeled by logical formulas involving `Cadbiom` model identifiers generated from BioPax files. As shown in Fig 3 in S1 Appendix, the computation of controllers in BioPax datasources relies on a pipeline involving several `Cadbiom` functions. First of all, the procedure *query design* is dedicated to the construction of the query. The features of a `Cadbiom` model built with `biopax2cadbiom` are exported with the `cadbiom-cmd` package. Three lists of internal identifiers can be generated, they correspond to (i) the complete list of biological compounds (ii) the list of - controllers - boundary compounds (iii) the list of genes. This command line also allows to generate mappings between internal identifiers and various databases thanks to the conservation of all cross-references with public databases (such as HGNC, Uniprot, Chebi) provided in the BioPAX files.

# 6 Trajectory analysis in the PID and ACSN Cadbiom models

The composition of the trajectories for a same gene can differ considerably between the PID and ACSN Cadbiom models. To illustrate this, we detailed the trajectories to activate the SPP1 gene in the two models (Fig. 4 in S1 Appendix ).

# References

[1] Türei D, Korcsmáros T, Saez-Rodriguez J. OmniPath: guidelines and gateway for literature-curated signaling pathway resources. Nat Methods. 2016;13(12):966–967.

[2] Kuperstein I, Bonnet E, Nguyen HA, Cohen D, Viara E, Grieco L, et al. Atlas of Cancer Signalling Network: A Systems Biology Resource for Integrative Analysis of Cancer Data with Google Maps. Oncogenesis. 2015;4:e160. doi:10.1038/oncsis.2015.19.

[3] Schaefer CF, Anthony K, Krupa S, Buchoff J, Day M, Hannay T, et al. PID: the Pathway Interaction Database. Nucleic Acids Res. 2009;37(Database issue):D674–679.

[4] Andrieux G, Le Borgne M, Théret N. An Integrative Modeling Framework Reveals Plasticity of TGF-$\beta$ Signaling. BMC Systems Biology. 2014;8:30. doi:10.1186/1752-0509-8-30.

[5] msoos. CryptoMiniSat 5.0.1 Released — with MIT License — Wonderings of a SAT Geek; 2010.

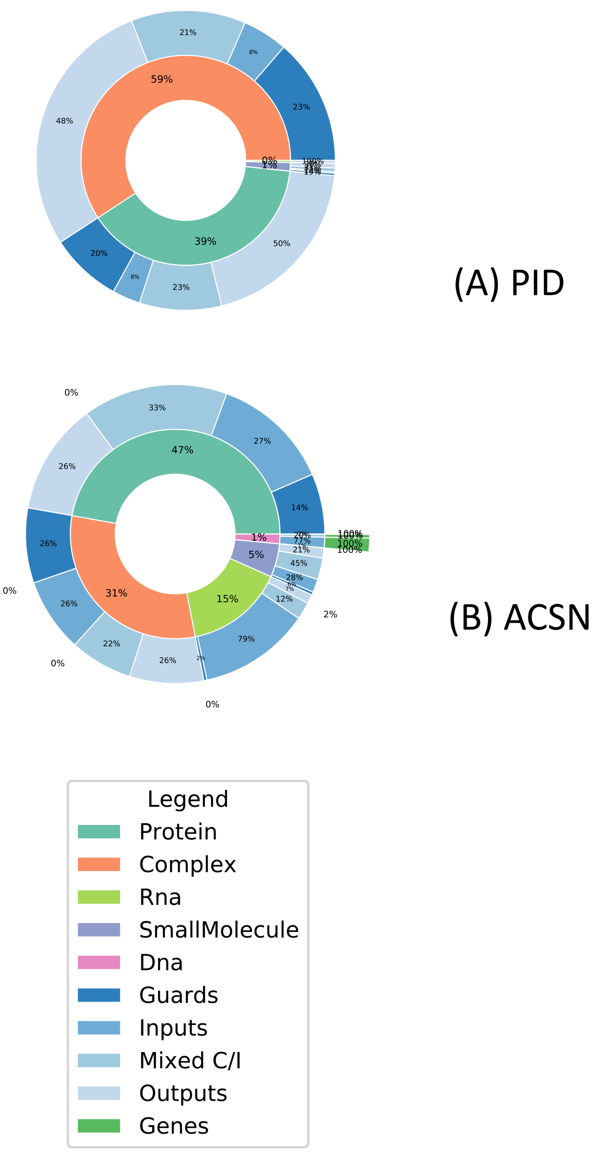


Figure 1: **BioPAX Classes from PID (A) and ACSN (B) databases and their corresponding role as boundary entities in the Cadbiom models.** The boundary entities are either pathway initiating molecules in the models considered, or controllers of these pathways whose production is not explained in the model. The inner circle of each figure describes the distribution of these boundary entities according to their BioPAX classes (protein, complex, small molecule, DNA, RNA). In the `Cadbiom` models, the genes are deduced from the identifiers of proteins, DNA and RNA, BioPAX entities. They are represented in the outer circle. For each class of BioPAX entities, the middle circle describes the role of the boundary entities in the `Cadbiom model`: either the molecule is an input of a transition in the model, or it is involved in the logical condition associated with a transition, or both.

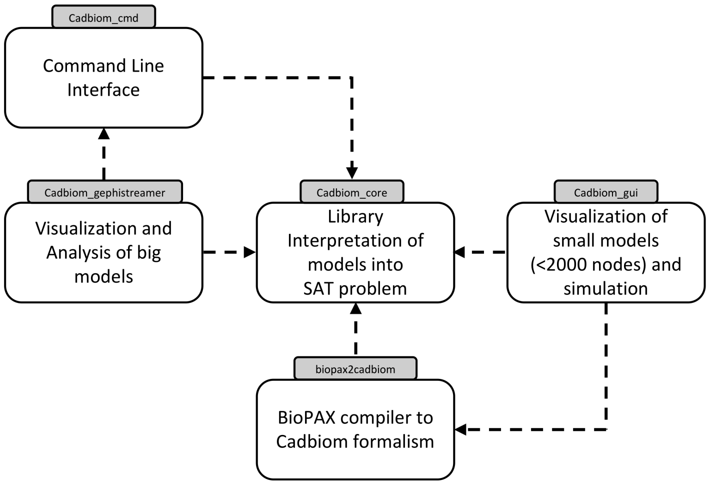


Figure 2: **UML package diagram of the `Cadbiom` suite.** The `Cadbiom` framework is divided into five Python packages: `cadbiom-gui` (Graphical User Interface), `cadbiom-core` (library), `cadbiom-cmd` (command line), `biopax2cadbiom` (conversion of the BioPAX models into the `Cadbiom` models) and `cadbiom-gephistreamer` (data analysis and visualization)

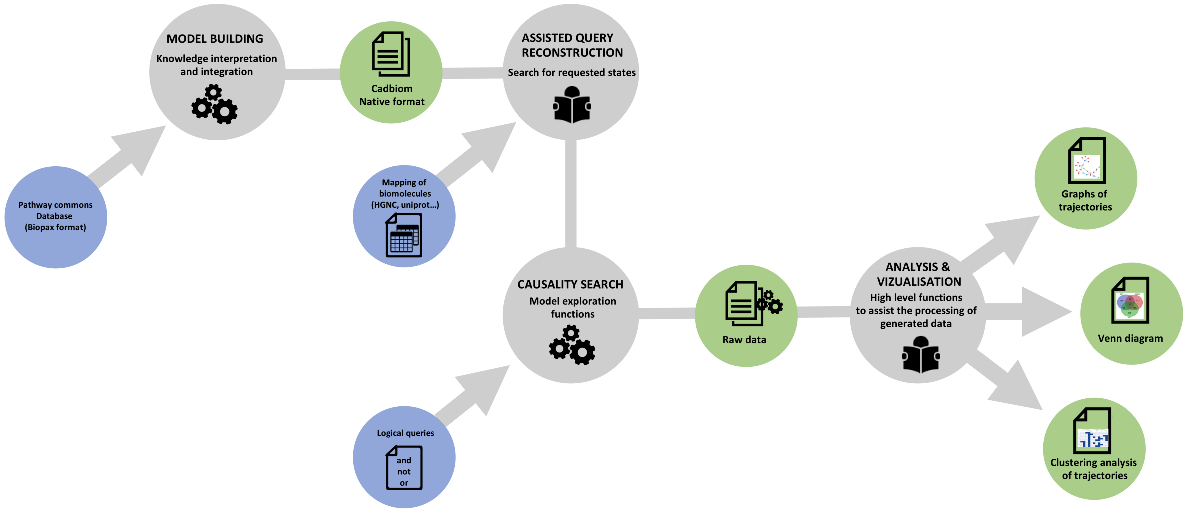


Figure 3: **Workflow of analysis of BioPAX resources with the `Cadbiom` suite** The different components of the `Cadbiom` suite (see Fig. 2) can be combined to perform a complete analysis of a BioPAX resource. First, a `Cadbiom` model can be automatically built to interpret automatically the content of a resource described with the BioPAX format (by using `biopax2cadbiom` ). Some resource models are already pre-computed and can be used directly. Then, based on a dictionary mapping `Cadbiom` biomolecule identifiers to uniprot, the user can build a query describing the expected phenotype to study. The search for controllers and trajectories is then run with the `cadbiom-cmd`. Finally, the user can analyze and visualize the results of the controller computation according to several views (graph of trajectories, Venn diagram, Clustering of trajectories), with the `cadbiom-cmd` and `cadbiom-gui` packages.

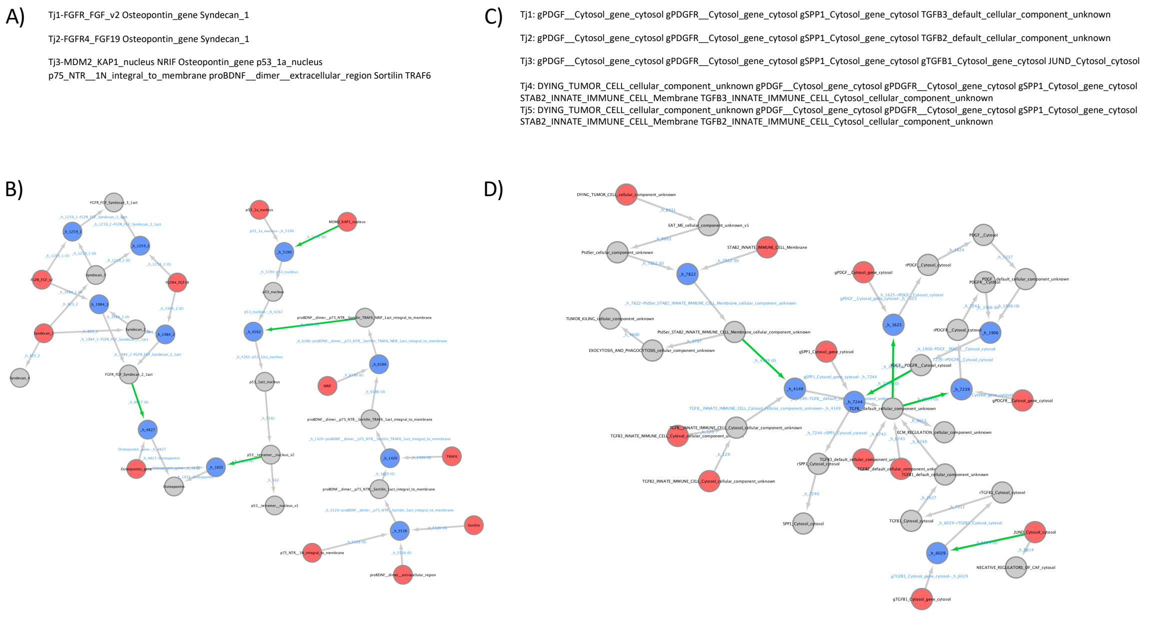


Figure 4: **Comparison of the trajectories to activate the SPP1 gene in the PID and ACSN `Cadbiom` models**. A) and C) list the composition of the trajectories for the PID and ACSN models, respectively. B) and D) are graphical representations of trajectories for the PID and ACSN models, respectively. Red nodes are cadbiom model boundaries of the model. Grey nodes are basic entities/intermediate molecules which are not at the periphery of the model. Blue nodes denote reaction in which there are more than one reagent or one reactant (many-to-many or one-to-many relationship between reactants). Grey arrows are unary reactions (one-to-one relationship). Green arrows are activations (control reactions).
