# Supplementary material for: Discrete modeling for integration and analysis of large-scale signaling networks
Source: PLoS Comput Biol. 2022 Jun 13;18(6):e1010175. doi: 10.1371/journal.pcbi.1010175 (PMC9232147; doi:10.1371/journal.pcbi.1010175)
Supplement: S1 Table — (PDF) [file pcbi.1010175.s002.pdf]

S1-Table: List of genes in PID and ACSN models (HUGO gene symbol)

PID	ACSN
A2M	ABCB1
ABCA1	ACT2
ABCA3	ACTA2
ABCB1	ADAM10
ABCC8	AIFM1
ABCG2	AKT1
ACADM	AKT2
ACADVL	ALAS1
ACHE	ALDOA
ACTA1	ALOX15
ACTA2	ANPT1
ADA	ANPT2
ADM	APAF1
ADORA2A	APBA1
ADORA2B	ARC
ADRM1	AREG
ADRP	ARF1
AEN	ARG1
AFP	ARG2
AGT	ATF3
AID	ATF4
AKT3	ATG10
ALAS	ATG12
ALB	ATG16L1
ALDH9A1	ATG16L2
ALDOA	ATG4A
ALDOB	ATG4B
ALOX12B	ATG4C
ALOX15	ATG4D
AMID	ATG5
ANF	ATG9A
ANGPT1	ATP2A2
ANGPT2	ATP5A1
AP1B1	ATP5B
APAF1	ATP5C1
APC	ATP5G1
APN	AXIN1
APOA1	AXIN2
ApoB	BAD
ARG1	BAG6
ARID3A	BAK1
ARNTL	BAX
ASCL1	BBC3
ATF3	BCL2
ATM	BCL2A1

ATP2B1	BCL2L1
ATP5PF	BCL2L11
AURKB	BCL3
Axin2	BCL6
AXL	BCL9
B-Myb	BECN1
BAK	BID
BAX	BIK
BBC3	BIRC2
BCAT1	BIRC3
BCL2	BIRC5
BCL2L1	BMI1
BCL2L14	BMP4
BCL6	BNIP3
BDH1	BRCA1
BDKRB2	BTLA
BGLAP	BUB1
BHLHE41	CA9
BID	CALM1
BIM	CASK
BIRC3	CASP10
BIRC5	CASP3
BMI1	CASP7
BNIP3	CASP8
BNIP3L	CASP9
BRCA1	CASR
BRCA2	CBLB
BRD2	CCL18
BTG2	CCL19
C3	CCL2
C4BPB	CCL20
CA1	CCL24
CA9	CCL26
CACNA1C	CCL3
CACNA1G	CCL5
CAD	CCL7
CAMK4	CCNA1
CASP1	CCNB1
CASP10	CCND1
CASP2	CCND2
CASP3	CCND3
CASP6	CCNE1
CASP7	CCNE2
CAT	CCR2
CAV1	CCR7
CBFB	CD160
CBLB	CD163
CBX5	CD1A

CCL11	CD209
CCL17	CD36
CCL2	CD40
CCL26	CD80
CCL3	CD86
CCL4	CDC20
CCL5	CDC20B
CCN1	CDC25A
CCNA1	CDC42
CCNB1	CDH1
CCNB2	CDH2
CCND1	CDH3
CCND2	CDH4
CCND3	CDK2
CCNE1	CDK4
CCNG1	CDK6
CCNK	CDKN1A
CCR5	CDKN1B
CD34	CDKN2A
CD3E	CDKN2B
CD4	CDKN2D
CD40LG	CEACAM20
CD82	CEBPB
CDC25A	CEBPD
CDC25B	CFLAR
CDC6	CHEK1
CDH1	CHOP
CDK1	CIB1
CDK4	CIITA
CDK5R1	CITED2
CDK6	CK2
CDKN1A	CLDN1
CDKN1B	CLDN2
CDKN2A	CLDN3
CDKN2B	CLDN4
CDKN2C	CLDN7
CDX1	CLL4
CDX4	CNN1
CEBPA	COL1A2
CEBPB	COX4I2
CEBPD	COX5A
CENPA	COX5B
CENPB	COX6A1
CENPF	COX7A2
CENPX	CRB3
CES1	CRELD2
CES2	CSF1R
CES3	CSF2

CES4A	CSF2RA
CES5A	CSF3
CFLAR	Csk
CGA	CST3
CHRNA1	CTGF
CHRNE	CTLA4
CHUK	CTNNB1
CISH	CTSB
CITED2	CTSD
CKM	CXCL1
CKS1B	CXCL10
CLCA2	CXCL12
CLOCK	CXCL16
CLTA	CXCL2
CLU	CXCL5
COL18A1	CXCL7
COL1A1	CXCL8
COL1A2	CXCR1
COL24A1	CXCR2
COL3A1	CXCR4
COL5A1	CYBB
COP1	CYCS
COPA	CYP1B1
CP	DDB2
CPT1A	DDIT4
CPT1B	DERL3
CRP	DKK1
CRY1	DKK3
CRY2	DNA2
CSDE1	DNAJB11
CSF1R	DNAJC3
CSF2	DNAM1
CSN2	DRAM1
CSRP2	DSC2
CTLA4	DSP
CTSD	DTX1
CX3CL1	DUSP1
CXCL1	DVL1
CXCL12	DVL2
CXCL9	DVL3
CXCR4	E2F2
CYC1	E2F3
CYP2C18	EDEM1
CYSLTR1	EDN1
CYSLTR2	EGF
DAPK1	EGFR
DCN	EGLN1
DCP1B	EGR1

DDB2	ELN3
DDIT3	EMILIN2
DDIT4	ENTPD1
DDX18	ENTPD2
DEDD	ENTPD3
DGKA	ENTPD5
DHFR	ENTPD6
DHRS3	EP300
DICER1	EPAS1
DKC1	ERE
DKK1	ERN1
DKK4	ERO1A
DLEU1	ESRRA
DLEU2	ETS1
DLK1	ETS2
DLX5	EZH1
DLX6	EZH2
Dmp1	F11R
DMTF1	F2RL2
DNTT	FAF1
DSCAM	FAP
DST	FAR1
DUSP1	FAS
DUSP5	FASL
DUSP6	FASLG
DUSP8	FCER2
E2F1	FECH
E2F2	FGF1
E2F3	FGF2
EBAG9	FGF7
EDN1	FLT1
EDN2	FLT3
EFNA1	FN1
EGFR	FOS
EGLN1	FOXA2
EGLN3	FOXC2
EGR1	FOXE1
EGR2	FOXF1
EGR3	FOXL1
eIF2A	FOXM1
eIF4A1	FOXO1
eIF4E	FOXO3
eIF4G1	FOXO4
ELAC2	FOXO6
ELANE	FOXP3
ELF2	FOXQ1
ENG	FTH1
ENO1	FZD1

EOMES	FZD3
EPHA2	FZD4
EPO	FZD7
EPOR	FZD8
ErbB2	G0S2
ESR1	GABARAPL1
ETS1	GADD45A
EVPL	GADD45B
F2	GADD45G
FABP4	GALNT3
FAS	GAPDH
FASLG	GATA1
FASN	GCLC
FBXO32	GCLM
FCER2	GCN4
FCGR1A	GJB2
FDXR	GJB3
FECH	GLI1
FGF4	GLI2
FGG	GLI3
FHL2	GLS
FIZZ1	GLS2
FLOT2	GPC6
FLT1	GPI
FOS	GPT2
FOSL1	GSK3B
FOSL2	GZMA
FOXA1	GZMB
FOXA2	HDAC1
FOXO3A	HDAC2
FOXP3	HERPUD1
FTH1	HES1
Furin	HEY1
FXN	HEY2
G6PC	HGF
GAA	HIF1A
GADD45A	HIP1
GADD45B	HIP1R
GADD45G	HK2
GAPDH	HLA-A
GAS1	HLA-B
GATA1	HLA-DRA
GATA3	HLX
GBP3	HMOX1
GCG	HOXA1
GCK	HR
GDF15	HRK
GHR	HSP60

GJA1	HSP90AA1
GLI1	HSP90B1
GLUT1	HSPA1A
GLUT2	HSPA1B
GLUT3	HSPA5
GPAM	HYOU1
GPX1	ICAM1
GPX2	ICOS
GRAMD4	ID1
GREB1	ID2
GSC	ID3
GSTM1	IDH3A
GTF2H2	IDO1
GZMA	IFN
GZMB	IFNA1
H2AZ1	IFNB
H2BC1	IFNB1
HADH	IFNG
HAGH	IGF1
HBG1	IGF2
HBG2	IGF2R
HBP1	IGFBP1
HES1	IGFBP3
Hes5	IGFBP6
HEY1	IL10
HEY2	IL11
HGF	IL12A
HIC1	IL12B
HK1	IL12RB2
HK2	IL13
HLA-A	IL13RA1
HLA-B	IL13RA2
HLA-DRA	IL15
HMGA1	IL15RA
HMGCS1	IL18
HMGCS2	IL18R1
HMOX1	IL18RAP
HNF4A	IL1A
HRK	IL1B
HSF1	IL1R2
HSP90AA1	IL1RN
HSP90B1	IL2
HSPA1A	IL23A
HSPA5	IL2RA
HSPA8	IL2RB
HSPD1	IL4
HTT	IL4R
ICAM1	IL5

ID1	IL6
ID2	IL6R
IFNB1	IL6ST
IFNG	ILK
IGF2BP1	INFB1
IGFBP1	INFG
IGFBP3	INHBA
IGHA1	INK4
IGHE	INPP5D
IGHG3	IRAK3
IGHG4	IREB2
IKBKB	IRF1
IKZF1	IRF4
IKZF2	IRF5
IKZF3	IRF8
IKZF4	IRF9
IKZF5	IRS2
IL10	ITA10
IL12A	ITB1
IL12RB2	ITB4
IL13	ITGA1
IL13RA2	ITGA11
IL17	ITGA2
IL17F	ITGA2B
IL18	ITGA3
IL18R1	ITGA4
IL18RAP	ITGA5
IL19	ITGA6
IL1A	ITGA7
IL1B	ITGA8
IL1R1	ITGA9
IL1RAP	ITGAE
IL2	ITGAL
IL23A	ITGAM
IL23R	ITGAV
IL24	ITGAX
IL27RA	ITGB1
IL2RA	ITGB2
IL2RG	ITGB3
IL3	ITGB5
IL4	ITGB6
IL4R	ITGB7
IL5	ITGB8
IL6	JAG2
IL8	JAK1
ILK	JUN
INS	JUNB
IQGAP1	JUP

IRF1	KCNN4
IRF4	KDM6B
IRF5	KDR
IRF8	KFRT1
IRF9	KLF4
IRP2	KLRB1
ITCH	KLRG1
ITGA3	KLRK1
ITGA6	KRT14
ITGB1	KRT18
ITGB2	KRT19
ITGB3	LAG3
ITGB4	LALS3
ITGB5	LCP2
IVL	LDHA
JAG1	LEF1
JAG2	LEO1
JAK1	LGALS3
JPO1	LIF
JUN	LIN7C
JUNB	LONP1
KCNIP4	LOX
KCNJ11	LOXL1
KDR	LRDD
KIR3DL1	LRP6
KIT	LTB
KLK2	LY96
KLK3	MAD2L2
KLRC3	MAF
KRT1	MANF
KRT14	MAOA
KRT17	MAPK1
KRT5	MAPK14
KSR1	MAPK3
LAMA1	MAPK6
LAMA3	MARCH1
LAMA4	MCL1
LBP	MCM7
LDHA	MDM2
LEF1	MEF2C
LEP	MICA
LGALS1	MICB
LIF	MIF
LIN28B	MIR1-1
lnc-RORA-4	MIR106B
LPL	MIR1245A
LSH	MIR132
LTA	MIR149

LYZ	MIR150
MAD1L1	MIR155
MAD2L1	MIR15A
MAP4K4	MIR15B
MAT2A	MIR16-1
MCL1	MIR16-2
MCM3	MIR17
MCM4	MIR17HG
MDM2	miR182
MEF2C	MIR183
MET	MIR185
MFGE8	MIR192
Mgp	MIR19A
MIR130B	MIR200A
MIR141	MIR203A
MIR146A	MIR204
MIR17	MIR205
MIR200A	MIR21
MIR22	MIR211
MIR23B	MIR212
MIR26A1	MIR221
MIR26A2	MIR223
MIR26B	MIR23A
MIR338	MIR23B
MIR34A	MIR27A
MIR429	MIR29A
MIR9-3	MIR29B
MIRLET7G	MIR300A
MITF	MIR300B
MLH1	MIR300C
MMP1	MIR302C
MMP14	MIR30C2
MMP2	MIR30E
MMP7	MIR31
MMP9	MIR320A
MPO	MIR34A
Mre11	MIR34B
MSH2	MIR34C
MT2A	MIR372
MTA1	MIR373
MTDH	MIR374A
MXD4	MIR375
Myb	MIR378
MYC	MIR424
MYCT1	MIR449A
MYF6	MIR455
MYL1	MIR493
NBN	MIR495

NCL	miR96
NDRG1	MMP1
NDRG2	MMP10
NDUFAF2	MMP11
NDUFS2	MMP13
NDUFV3	MMP14
NEDD4L	MMP2
NEK2	MMP3
NEUROG1	MMP7
NEUROG3	MMP9
NFATC1	MPP5
NFATC3	MPP6
NFKBIA	MRC1
NKX2-1	MRC2
NKX3-1	MRPS12
NKX3-2	MYB
NLRC4	MYC
NME1	MYCN
NME2	MYD88
NOS2	MYL12A
NOS3	NCF1
NOTCH1	NCR1
NOXA	NCR2
NPM1	NCR3
NR1D1	NCR3LG1
NR1I3	NDUFA4L2
NR3C1	NDUFAF2
NR4A1	NDUFS8
NRAS	NFAT5
NRG1	NFATC1
NRIP1	NFATC2
NSG1	NFATC3
NT3	NFATC4
NT5E	NFKB1
NTRK1	NFKB2
NTS	NFKBIA
ODC	NFKBID
OGG1	NFKBIZ
ONECUT1	NKX2-2
OPRM1	NOS2
ORC1	NOX4
OSM	NOXA1
Pax6	NQO1
PCBP4	NRF1
PCK1	NUCB2
PCK2	OCLN
PCNA	ORMDL2
PDCD10	P4HTM

PDE9A	PANK1
PDGFRA	PARD6A
PDGFRB	PATJ
PDX1	PAX6
PEA15	PAX9
PEG10	PDCD1
PENK	PDGFA
PER1	PDGFB
PER2	PDGFC
PERP	PDGFD
PFKFB3	PDGFRA
PFKL	PDGFRB
PFKM	PDIA2
PGK1	PDIA3
PGM1	PDIA4
PGR	PDIA5
PIDD1	PDIA6
PIGR	PDK1
PIM1	PDK3
PISD	PDK4
PITX2	PERP
PKLR	PFKFB3
PKM	PFKFB4
PLA2G4A	PFKM
PLAU	PGK1
PLAUR	PHLDA3
PLK1	PHLP1
PLK3	PHLP2
PLPP1	PIGA
PML	PIK3CD
PMS2	PINK1
POLA1	PKM2
POLR3D	PKP3
POMC	PLAU
POU5F1	PLK1
PPARD	PMAIP1
PPARGC1A	POSTN
PPM1J	PPAR
PPP3CA	PPARC1B
PRDM1	PPARG
PRDM15	PPARGC1A
PRDX3	PPP1R15A
PRF1	PRDX1
PRKAB1	PRDX3
PRKCD	PRF1
PRL	PRKAB1
PRTN3	PRKAB2
PTCH1	PRKAR1A

PTCRA	PRKAR2A
PTEN	PRKCE
PTGDS	PROK1
PTGS2	PTCH1
PTHLH	PTCH2
PTMA	PTEN
PTP4A1	PTER2
PTPA	PTGS2
PTPN1	PTP4A3
PTPN2	PTPN1
PTPRK	PXN
PYCARD	PYCARD
RAB38	RAB5A
RAB7A	RAB5B
RAD51	RAB5C
RAG2	RAC1
RANBP1	RAC2
RB1	RAD51
RBBP4	RAP1A
RBBP8	RAP1B
RBL1	RB1
RBL2	RCHY1
RCAN1	REL
RCC1	RELA
RCHY1	RELB
RGCC	RHOA
RIOX2	RIPK1
RNF128	RRM2B
RNF144B	RUNX1
RNF43	RUNX2
RPL11	S100A4
RPS27L	S100A7
RRAD	S100A8
RRM1	S100A9
RRM2	SATB2
RRM2B	SCO2
RUNX1	SDF2L1
S100A2	SDHB
S100A7	SDHC
S1PR1	SDHD
SALL4	SEL1L
SCGB1A1	SERPINE1
SCN3B	SERPINF1
SEC14L2	SESN1
SELE	SESN2
SELP	SFRP1
SERPINA1	SH2D1A
SERPINB5	SIAH1

SERPINE1	SIN3A
SERPINI1	SIRT1
SESN1	SIVA1
SFI1	SIX1
SFN	SIX3
SFRP1	SLC16A4
SFTPA1	SLC1A5
SFTPA2	SLC2A1
SFTPD	SLC2A4
SFXN3	SLC38A5
SGK1	SLC7A11
SH2D1A	SMAD1
SHH	SMAD2
SHMT1	SMAD3
SIRT1	SMAD4
SKP2	SMAD5
SLC11A1	SMAD6
SLC11A2	SMAD7
SLC1A5	SMAD9
SLC22A2	SNAI1
SLC25A3	SNAI2
SLC3A2	SOCS1
SLUG	SOCS2
SMAD7	SOCS3
SMARCC1	SOD1
SMARCD3	SOD2
SNAIL	SOX2
SOCS1	SOX4
SOCS3	SPI1
SOD1	SPP1
SOD2	SRXN1
SP5	STAB1
SPATA18	STAT1
SPP1	STAT3
SREBF1	STAT4
SRF	STAT5A
SRP9	STAT6
STEAP3	STC1
TAF4B	STMN1
TAGLN	STMN2
TAP1	STMN3
TAT	STMN4
TBX21	SURF1
TBXT	SYNV1
TCF3	TAGLN2
TCF7	TBX2
TCF7L2	TBX21
TEK	TCF3

TERT	TCF4
TF	TDGF1
TFAP2C	TERC
TFEC	TERT
TFF1	TFAM
TFF3	TFB1M
TFRC	TFB2M
TGFA	TFGB3
TGFB2	TFRC
TGFBR2	TGFB1
THBD	TGFB2
THY1	TGFB3
TIGAR	TGFBR1
TIMELESS	TGFBR2
TIMP1	TGM1
TK1	TIGAR
TLE4	TIMP1
TLX2	TIMP2
TMEFF2	TIMP3
TMEM126A	TJP1
TMPRSS2	TJP2
TNF	TJP3
TNFAIP3	TKT
TNFRSF10A	TLE1
TNFRSF10B	TLR2
TNFRSF10C	TLR4
TNFRSF10D	TNC
TNFRSF18	TNF
TNFRSF4	TNFAIP3
TNFRSF9	TNFRSF10A
TNFSF11	TNFRSF10B
TOM1	TNFRSF18
TOP2A	TNFRSF1A
TP53	TNFRSF1B
TP53AIP1	TNFRSF4
TP53I3	TNFSF10
TP53INP1	TNIP3
TP63	TP53
TP73	TPI1
TRAF4	TRADD
TRD	TRIB3
TRIAP1	TSC2
TRPC6	TSLP
TRPV1	TWIST1
TSC2	TWIST2
TTR	TXN
TUBA1A	TXNIP
TWIST1	TXNRD1

TYRP1	TYROBP
UBTF	UBE2B
UCP2	UDLP1
UXT	UDLP3
VCAM1	ULBP2
VCAN	ULK1
VCL	ULK2
VDR	UQCRB
VEGFA	USP1
VIPR1	UVRA
VTLGL1	VDAC1
VTN	VDAC3
WASF1	VEGFA
WNT5A	VEGFB
WWP1	VEGFC
XBP1	VEGFD
XRCC1	VIM
YAP1	WIF1
YEATS4	WNT1
YWHAG	WNT11
YWHAQ	WNT2B
ZCCHC12	WNT3
ZEB1	WNT5A
ZFAND5	WRN
ZFP36	XBP1
ZFP36L1	XIAP
	XPC
	YWHAE
	YY1
	ZEB1
	ZEB2
